# Highly Bioadaptable Hybrid Conduits with Spatially Bidirectional Structure for Precision Nerve Fiber Regeneration via Gene Therapy

**DOI:** 10.1002/advs.202309306

**Published:** 2024-03-14

**Authors:** Renliang Zhao, Xiangtian Deng, Jizhao Dong, Chen Liang, Xiaozhong Yang, Yunfeng Tang, Juan Du, Zilu Ge, Dong Wang, Yifan Shen, Lianghua Jiang, Wei Lin, Tonghe Zhu, Guanglin Wang

**Affiliations:** ^1^ Orthopedics Research Institute Department of Orthopedics West China Hospital Sichuan University Chengdu 610041 P. R. China; ^2^ Trauma Medical Center Department of Orthopedics Surgery West China Hospital Sichuan University Chengdu 610041 China; ^3^ Multidisciplinary Centre for Advanced Materials Institute for Frontier Medical Technology School of Chemistry and Chemical Engineering Shanghai University of Engineering Science 333 Longteng Rd. Shanghai 201620 P. R. China; ^4^ Head & Neck Oncology Ward Cancer Center West China Hospital Cancer Center Sichuan University Chengdu 610041 P. R. China; ^5^ Spine Lab Department of Orthopedic Surgery The First Affiliated Hospital Zhejiang University School of Medicine Hangzhou 310003 China; ^6^ Department of Orthopedic Trauma The First People's Hospital of Kunshan affiliated with Jiangsu University Suzhou Jiangsu 215300 P. R. China; ^7^ Department of Gynecology West China Second Hospital Sichuan University Chengdu 610041 P. R. China

**Keywords:** genetic therapy, nerve conduits, neuroautonomous selectivity, neuroplasticity, Wallerian degeneration

## Abstract

Peripheral nerve deficits give rise to motor and sensory impairments within the limb. The clinical restoration of extensive segmental nerve defects through autologous nerve transplantation often encounters challenges such as axonal mismatch and suboptimal functional recovery. These issues may stem from the limited regenerative capacity of proximal axons and the subsequent Wallerian degeneration of distal axons. To achieve the integration of sensory and motor functions, a spatially differential plasmid DNA (pDNA) dual‐delivery nanohydrogel conduit scaffold is devised. This innovative scaffold facilitates the localized administration of the transforming growth factor β (TGF‐β) gene in the proximal region to accelerate nerve regeneration, while simultaneously delivering nicotinamide mononucleotide adenylyltransferase 2 (NMNAT2) to the distal region to mitigate Wallerian degeneration. By promoting autonomous and selective alignment of nerve fiber gap sutures via structure design, the approach aims to achieve a harmonious unification of nerve regeneration, neuromotor function, and sensory recovery. It is anticipated that this groundbreaking technology will establish a robust platform for gene delivery in tissue engineering.

## Graphical abstract

This study introduces a spatially differential plasmid DNA dual‐delivery nano hydrogel conduit fabricated with HA‐GMA/PEI (HP) for restoring extensive segmental nerve defects. It utilizes transforming growth factor β(TGF‐β) delivery via HP (TGF‐β@HP) in the proximal region to enhance nerve regeneration and nicotinamide mononucleotide adenylyltransferase 2 (NMNAT2) via HP (NMNAT2@HP) in the distal region to prevent Wallerian degeneration. The design promotes selective nerve fiber alignment, aiming for improved neuromotor and sensory function recovery.

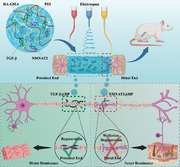

## 1 Introduction

Peripheral nerve injury (PNI) is frequently instigated by momentous public health crises, such as traffic collisions, earthquakes, and explosions, and is accompanied by impaired limb function.^[^ ^1^, ^2^ ^]^ PNI can culminate in an incomplete or complete loss of sensory, motor, and autonomic functions, severely impinging on the patient's quality of life. After the injury, the distal nerve stump undergoes Wallerian degeneration, whereby both axons and myelin sheaths disintegrate, and the detritus is scavenged by phagocytosis of Schwann cells (SCs) and macrophages.^[^ ^3^, ^4^ ^]^ Additionally, a series of changes (such as chromatin breakdown) occur in the cytoplasm of the broken axon, in preparation for its repair.^[^ ^5^ ^]^ Neurotrophic growth factors, including neural growth factor (NGF), are known to facilitate the regeneration of injured axons.^[^ ^6^ ^]^ Nonetheless, although the neuroectoderm can uphold local concentrations of neurotrophic factor, its source and expression are transient, and they fail to effectively foster the restoration of substantial lengths of nerve axons.^[^ ^7^, ^8^, ^9^ ^]^

Following nerve injury, the proximal axon sprouts to create a “growth cone” that recognizes biological signals and neurotrophic factors, thereby determining the direction of axon growth during regeneration.^[^ ^10^, ^11^ ^]^ The distal axon, on the other hand, undergoes degeneration, with the damaged axon and its surrounding myelin sheath experiencing retrograde degeneration.^[^ ^12^ ^]^ After 48–96 h, nerve fibers, axons, and myelin sheaths undergo “Wallerian degeneration” and are subsequently cleared out by phagocytosis of SCs and macrophages, leaving the entire distal nerve canal in an “empty” state. Studies have revealed that within 7 days of injury, nerve fibers in the endometrial canal of the transected nerve display “neuroautonomous selectivity,” where fibers of the same type (sensory or motor) are attracted to each other, guiding the nerve fibers to properly match the injured area with the target organ under their control.^[^ ^13^, ^14^ ^]^ However, the expression of neurotrophic factors is usually transient, and large segments of nerve defects do not allow for effective “nerve fiber auto selectivity,” leading to difficulties in regenerating motor and sensory axons to find their original intraneuronal pathways.^[^ ^15^ ^]^ Prolonged denervation of SCs and muscle fibers may result in a loss of their growth support phenotype and decreased numbers.^[^ ^16^ ^]^ Therefore, restoring nerve continuity and sensory function transport requires not only promoting the regenerative capacity of the proximal end but also reasonably controlling the degeneration of the distal axons. Catheters endowed with multifunctional capabilities and biomimetic architectures have already garnered considerable success across various domains of tissue engineering, including neural, vascular, and other tissue systems.^[^ ^17^ ^]^ Bionic nerve conduits should emulate the normal growth environment of nerve fibers while maintaining the required cytokines for nerve growth in space and time, preserving the anatomical structure of nerve fibers. Restricted proximal regeneration and unstoppable distal Wallerian degeneration are the main causes of limited sensory and motor function recovery after nerve repair, highlighting the need to comprehend the molecular basis of repair processes in PNI and incorporate these specific processes into new nerve guidance conduits (NGCs).

Gene therapy has demonstrated significant effectiveness in peripheral nerve studies, enhancing nerve growth, mitigating inflammatory responses, and improving neuropathological conditions through precise gene delivery mechanisms. DNA can be administered via viral or non‐viral methods, with hydrogels serving dual roles: as a scaffold for cell culture and as a protective medium for gene transport, safeguarding DNA against degradation and ensuring its successful integration into cells, thanks to their advantageous, matrix‐like configuration. Scholars have employed hydrogels to transport DNA, siRNA, and miRNA, orchestrating the regenerative processes of peripheral nerves and the spinal cord, and facilitating the myelination of nerve fibers.^[^ ^18^, ^19^ ^]^

The high expression of suppressor of cytokine signaling 3 (SOCS3) at the site of nerve injury hinders neuronal plasticity and regenerative capacity by suppressing the nerve growth factor pathway,^[^ ^20^ ^]^ the neuroinhibitory factor SOCS3 was found to affect the repair process after neurological injury by inhibiting macrophage M2‐type polarization, blocking nerve growth factor secretion, and limiting axonal growth and nerve regeneration. TGF‐β plays a crucial role in the nervous system's development and activates the intrinsic growth capacity of neurons and neurovascular coupling by regulating immune cells.^[^ ^21^ ^]^ It has been reported that TGF‐β’s neuroprotective effect may function by modulating SOCS3's inhibitory impact, and TGF‐β application can further promote the regenerative ability of peripheral nerve axons.^[^ ^22^ ^]^ Furthermore, distal nerve axon injury can cause Wallerian degeneration due to the accumulation of nicotinamide mononucleotide (NMN).^[^ ^23^, ^24^, ^25^ ^]^ The toxic effect of NMN induces axonal NAD+ depletion, and the NMN/NAD+ ratio increases, promoting the degenerative process of Wallerian degeneration. NMNAT2, produced specifically by the neurocyte, can convert NMN to NAD+ and thus maintain axonal NAD+ levels, which is important for slowing Wallerian degeneration and maintaining axonal function in the distal end of the injured axon.^[^ ^26^, ^27^, ^28^ ^]^ Therefore, in this study, we fabricated a spatially differential pDNA dual‐delivery nanohydrogel conduit scaffold with TGF‐β and NMNAT2 delivery. This strategy, promoting nerve fiber regeneration proximally and inhibiting axonal apoptotic degeneration distally, can transform the clinical problem of large segmental nerve defects into nerve transection injuries. Therefore, incorporating this concept into NGC design is a novel and unreported NGC design strategy.

## 2 Results and Discussion

### 2.1 Fabrication and Characterization of Nerve Conduits

**Figure 1:**
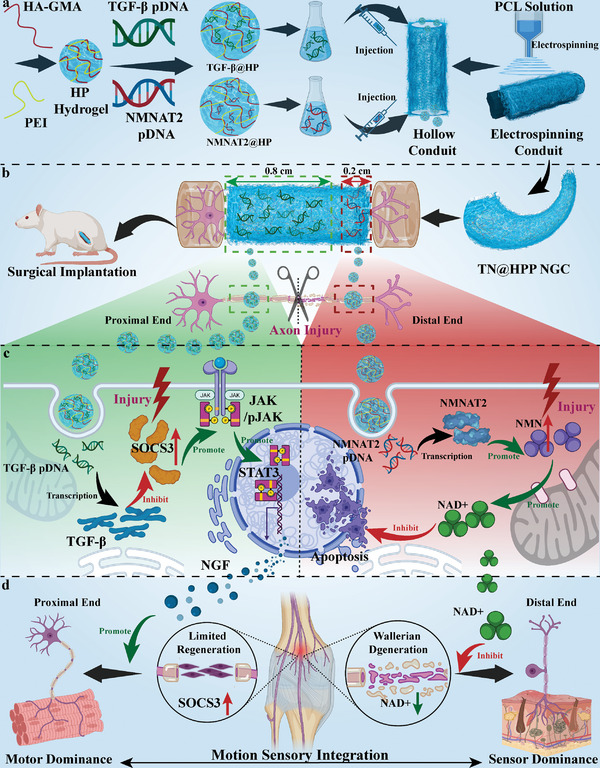
The schematic diagram of the spatially aligned composite catheter for nerve regeneration through gene therapy achieves a unification of nerve growth and function.

Most nerve conduits utilized clinically for large segmental nerve defects are made of polymeric materials and possess a single‐lumen structure.^[^ ^29^, ^30^ ^]^ While this structure does enhance the regenerative capacity of nerves to a certain extent, it results in poor recovery of nerve function for the patient.^[^ ^31^ ^]^ Multichannel, gel‐filled, and multi‐hollow nerve conduits have shown better potential in providing nutrients and a microenvironment conducive to nerve growth when compared to hollow nerve conduits.^[^ ^32^ ^]^ However, despite these strategies simulating the anatomical structure of nerve fibers, they are unable to imitate the dynamic changes involved in nerve regeneration or the differential distribution of nerve growth factors. Therefore, while the continuity of nerve fibers may be restored, both sensory and motor functions remain unsatisfactory.^[^ ^33^ ^]^ Our study entailed the design and preparation of nano hydrogel‐filled electrospun nerve catheters. These catheters were created to replicate the porous fiber structure of nerve fibers that are encapsulated by an outer membrane and possess an inner, gel‐filled, aqueous extracellular matrix survival microenvironment, the synthesis strategy is shown in the diagram (**Figure** **1**). Hydrogels capable of delivering pDNA of TGF‐β and NMNAT2 were infused into the proximal and distal ends of the catheter, respectively (Figure S1, Supporting Information). This approach mimicked the spatially differentially distributed cytokines involved in nerve axon regeneration, resulting in the autonomous selectivity of peripheral nerve fibers. The schematic diagram represents the design strategy employed in this nerve conduit to achieve proximal nerve regeneration, as well as distal nerve apoptosis inhibition through spatially differentially distributed hydrogel perfusion. This approach transformed the therapeutic dilemma of large segmental nerve defects into a strategy of transecting nerve gap sutures, ultimately leading to the unity of nerve regeneration and functional recovery.

**Figure 2:**
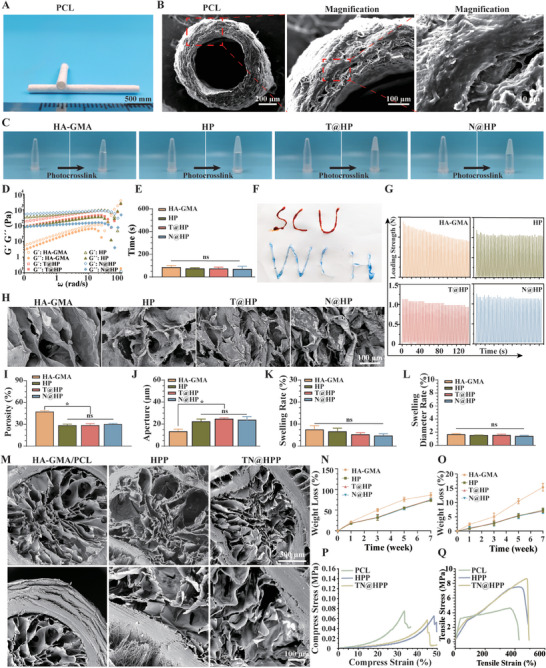
Exploration of the Structural and Functional Attributes of a Composite Neural Guidance Channel (NGC). A) A visual examination reveals the hollow structure of the electrospun neural conduit, showcasing both its longitudinal and cross‐sectional profiles. B) Detailed cross‐sectional observations via scanning electron microscopy unveil the intricate architecture of hollow PCL neural conduits, with images capturing the nuances of the conduit walls at varied magnifications. C) Visualization of the photopolymerization process within hydrogels, encompassing HA‐GMA, the hybrid HA‐GMA/ PEI hydrogels (denoted as HP), which incorporate PEI into HA‐GMA, alongside HP hydrogels infused with transforming growth factor‐beta (TGF‐β) plasmid (T@HP) and nicotinamide mononucleotide adenylyltransferase 2 (NMNAT2) plasmids (N@HP). D) Rheological analysis delineates the viscoelastic properties of HA‐GMA, HP, T@HP, and N@HP hydrogels. E) The temporal dynamics of gelation for HA‐GMA, HP, T@HP, N@HP hydrogels. F) Evaluation of the injectability of HP hydrogels. G) Stress–strain relationships during compression cycles for HA‐GMA, HP, T@HP, N@HP hydrogels. H) Scanning electron microscopy offers a microscopic glance at the structural composition of HA‐GMA, HP, T@HP, N@HP hydrogels. I) Estimation of the porosity within HA‐GMA, HP, T@HP, N@HP hydrogels. J) Measurement of pore dimensions across HA‐GMA, HP, T@HP, N@HP hydrogels. K) Quantification of the swelling capacity of HA‐GMA, HP, T@HP, N@HP hydrogels. L) Rate of swelling diameter expansion for HA‐GMA, HP, T@HP, N@HP hydrogels. M) Cross‐sectional scanning electron microscopy of composite NGCs encapsulating HA‐GMA hydrogel within PCL (HA‐GMA/PCL), HP hydrogel within PCL (HPP), and TN@HP hydrogel within PCL (TN@HPP). N) Analysis of the degradation behavior of HA‐GMA, HP, T@HP, N@HP hydrogels in dithiothreitol (DTT). O) Investigation into the degradation patterns of HA‐GMA, HP, T@HP, N@HP hydrogels in phosphate‐buffered saline (PBS). P) Assessment of the compressive strength for NGCs comprised of PCL, HPP, TN@HPP. Q) Evaluation of the tensile strength for PCL, HPP, and TN@HPP constructs. (Data encapsulate the findings from independent studies; all presented figures are expressed as mean ± standard deviation, with *n* = 5; significance denoted by ★ *p* < 0.05).

Subsequently, we synthesized the nerve conduit of polycaprolactone (PCL) by coaxial electrospinning technique, and the appearance of the conduit was observed and analyzed. The appearance of the wall is wound with electrospun yarn, and the hollow structure can be observed in the cross‐section, and the inner and outer diameters can be observed to match the thickness of the nerve fibers and the thickness of the nerve outer membrane, respectively (**Figure** **2**A). We further evaluated the spinning morphology as well as the lumen structure of PCL nerve conduits by transmission electron microscopy (TEM) examination, and the walls of the hollow PCL catheter were wound with electrospun fibers, which could be observed in the walls and lumen (Figure 2B). Next, we were ready to prepare the hydrogel filled inside the catheter (as a group: including hyaluronic acid‐glycidyl methacrylate (HA‐GMA), HP hydrogels with polyethylenimine (PEI) added (HP), and gels with TGF‐β (T@HP) and NMNAT2 plasmids added (N@HP)). The general picture shows that the HA‐GMA hydrogel was crosslinked and fixed at the bottom of the glass vial after UV irradiation, and the addition of PEI, TGF‐β, and NMNAT2 plasmids did not affect the gel‐forming ability of the hydrogel (Figure 2C), and the rheological data of the hydrogel changed after crosslinking, The kinetics of hydrogel formation were assessed by evaluating *G*′ (energy storage modulus) and *G*′′ (loss modulus). As shown by the results, G′ exceeds G′′ and exhibits frequency‐dependent properties, indicating the formation of an elastic network (Figure 2D). The addition of PEI and plasmid did not change the hydrogel formation time (Figure 2E). The hydrogel is extruded using a syringe and the letters written indicate that the hydrogel is injectable (Figure 2F). Next, we further evaluated the mechanical properties of the hydrogels and examined their cyclic compression performance, and found that after 10 cycles of compression, the hydrogels were able to return to their normal properties, with dynamic self‐healing ability (Figure 2G). Next, the morphology of the hydrogels was observed by scanning electron microscope (SEM), and the results suggested that the hydrogels of different compositions had a multi‐hollow structure, and the appropriate pore structure was more favorable for the cells to grow in (Figure 2H), pore size and porosity of the four hydrogels, and the pore size decreased and porosity of the hydrogels increased after the addition of PEI, whereas the continued addition of different components of plasmids did not influence the pore size and porosity of the HA‐GMA/PEI (HP) hydrogels. The pore size and porosity of HP hydrogels were not affected by the addition of different plasmids (Figure 2I,J). Swelling rate, as well as swelling diameter, is the key factors affecting the application of hydrogels, the addition of PEI to HA‐GMA hydrogel did not increase its swelling rate and swelling diameter, and the addition of TGF‐β and NMNAT2 plasmid to the composite hydrogel did not significantly change its swelling rate and swelling diameter (Figure 2K,L). Next, we evaluated the degradation of the different hydrogels in vitro, the results showed that the hydrogels could be degraded in dithiothreitol (DTT) (Figure 2N), The presence of the reducing agent DTT accelerated the degradation of HA‐GMA, PEI increased and stabilized HA‐GMA, leading to a decrease in the rate of its degradation, and then the degradation of the various types of hydrogels in phosphate buffered saline (PBS) was low, at a steady rate in degradation (Figure 2O). Finally, we prepared an electrospun neural conduit filled with composite hydrogel and evaluated its SEM and mechanical strength. In the SEM results, it can be observed that the hollow electrospun conduit is filled with porous hydrogel (Figure 2M), which is used for the loading of relevant plasmids and the simulation of the structure of the growth microenvironment. The axial tensile stress and compressed stress curve can also be observed that the HA‐GMA hydrogel with added PEI can enhance the axial tensile strain and compress strain of the composite catheter (Figure 2P,Q), which can effectively cope with the nerve traction faced during the suture process and avoid catheter fracture and nerve traction injury.

### 2.2 Biocompatibility of SC on Electrospun

Despite the widespread use of nerve catheters for clinical treatment of nerve repair and regeneration, and their potential benefits, clinical practice is often complicated by local infections and allergies caused by excessive catheter retention, as well as nerve injury and catheter rupture caused by stiffness discomfort, all of which are due to immune reactions at the implantation site caused by implantable polymer materials.^[^ ^34^ ^]^ Furthermore, fibrosis due to inflammatory infiltration can severely affect the function of nerve regeneration.^[^ ^35^, ^36^ ^]^ Therefore, the clinical application of implantable biomaterials requires a rigorous biosafety assessment.

**Figure 3:**
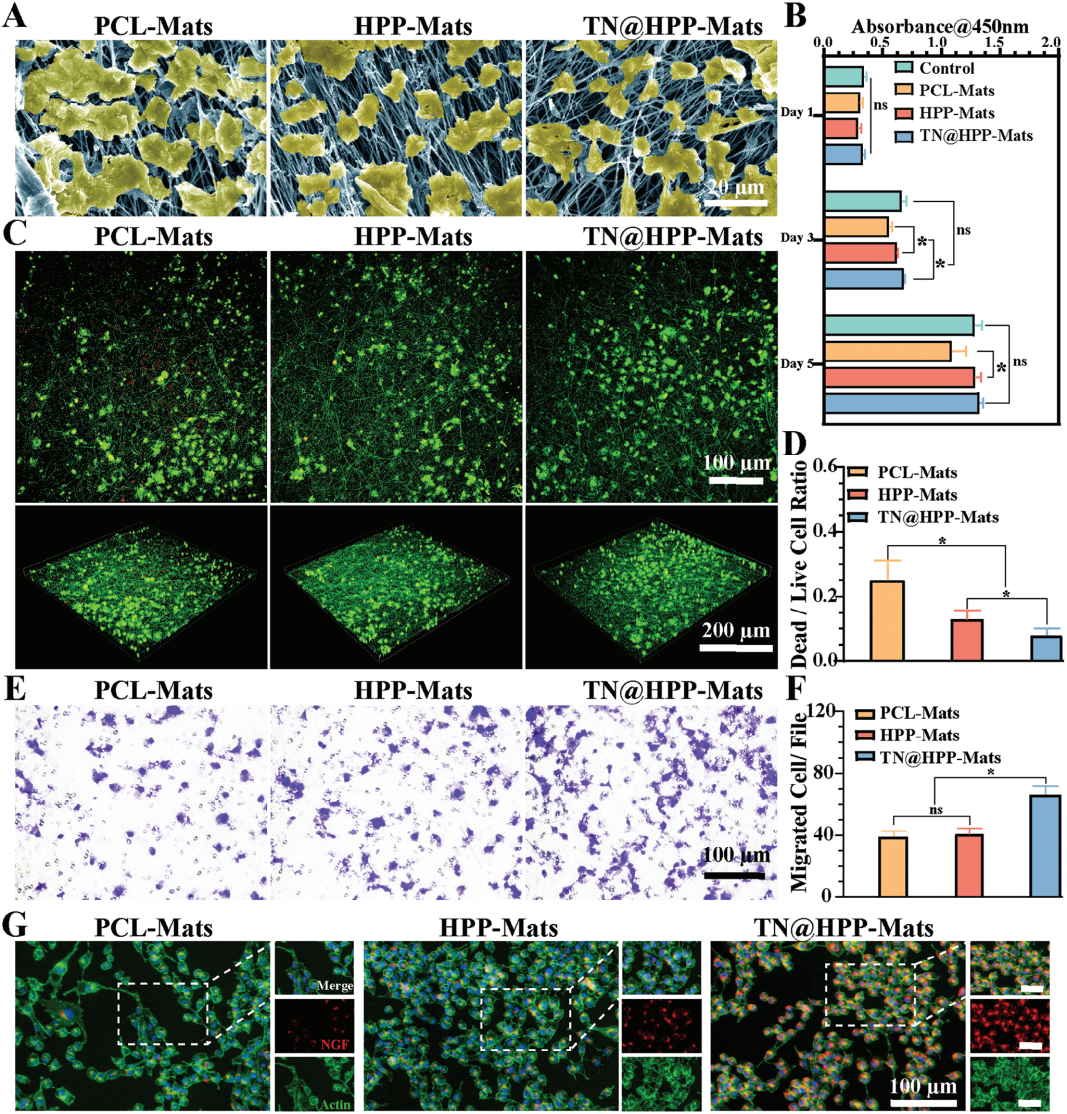
Evaluation of the biocompatibility of composite neural conduits. (A) Detailed examination through scanning electron microscopy revealed the adherence of cultured Schwann cells (SCs) to the surface of nanofibers. (B) The interaction between SCs and nanofibers was quantified using the colorimetric CCK‐8 assay, measuring cellular absorbance. C) The viability of neuronal cells on nanofibers was meticulously evaluated using live/dead staining; this technique allowed for the discernment of viable and nonviable cells on the fiber membranes, distinguished by Calcein/PI staining under confocal microscopy. D) The proportion of live and dead cells was statistically analyzed with ImageJ software, providing a quantitative assessment of cell viability. E) Post‐Transwell migration, SCs were marked with crystal violet, showcasing their migratory capacity across the membrane. F) Transwell migration data were analyzed quantitatively using ImageJ software, offering insights into the migratory behavior of Schwann cells. G) Immunofluorescence staining facilitated the investigation of nerve growth factor (NGF) expression in SCs residing on the fiber membrane; NGF was highlighted with red fluorescence, F‐actin with green, and cell nuclei with blue, providing a comprehensive view of cellular architecture and protein expression. (These findings, drawn from independent experiments, are presented as mean ± standard deviation, *n* = 5; significance denoted by ★ *p* < 0.05, with non‐significant results marked as ns).

In this study, we further evaluated the biosafety of composite catheters. Initially, we cultured cells on electrospun film and observed the morphological changes of SCs on the spun silk fiber film (PCL‐Mats: PCL electrospun film sheets in their singular state; HP‐Mats: film sheets soaked in the HA‐GMA/PEI gel; TN@HPP‐Mats: film sheets immersed in the HP gel infused with TGF‐β and NMNAT2 plasmids). The results indicated that a large number of SCs were attached to the electrospun fiber film with good cell morphology and obvious cell cytosol (**Figure** **3**A). Next, we evaluated cell survival on days 1, 3, and 5 using the CCK‐8 approach, respectively (Figure 3B). The results showed that the PCL electrospun fiber films had an inhibitory effect on SCs at days 3 and 5 compared to the control group. This may be related to mechanical damage to the cells, while no statistical difference could be observed in the composite catheter TN@HPP‐Mats group compared to the control group. Next, we observed the growth of SCs on the fibrous membrane using live‐dead staining (Figure 3C). The results showed that the morphology of fibrous membrane. SCs were clear and a small number of red‐labeled SCs were observed in the simple PCL control group as dead cells, while no dead red‐labeled SCs were observed in the composite group. The statistical analysis suggested that the mortality rate with SCs was significantly lower than that of the simple PCL control group (Figure 3D). In addition, we further examined the biological properties of the composites, including migration ability assessment and neural differentiation ability assessment. The results showed that the composite nerve fibers carrying pDNA significantly increased the migratory capacity of SC compared to the PCL‐Mats fiber film alone, with statistical results suggesting a statistical difference (Figure 3E,F). NGF is the earliest discovered and the most typical neurotrophic factor and the most important bioactive molecule affecting the nervous system, which not only has a trophic effect on normal nerve cells, but also plays a regulatory role in nerve repair to maintain the survival and function of nerve cells, promotes nerve cell differentiation and axon extension, and is therefore essential for PNI repair. It could also be observed that the composite nerve conduit significantly increased the expression level of NGF in SC cells, with more red‐labeled NGF expression observed in the graph (Figure 3G and Figure S2, Supporting Information). All of these results suggest that composite nerve conduits have better biosafety and pro‐neural differentiation ability.

### 2.3 TGF‐β@HP Promotes Nerve Regeneration via Activation of the JAK1/STAT3 Pathway

Compared to the central nervous system, peripheral nerves possess a certain degree of regenerative capability.^[^ ^37^, ^38^ ^]^ Myelin cells and SCs secrete cytotoxic factors to encourage axon regeneration after injury. The regeneration of nerves is influenced by factors such as the distance between the injury site and the cytosol, the concentration of neurotrophic factors, and the expression of intrinsic nerve genes.^[^ ^39^ ^]^ Severe transection injuries impede the healing ability of nerve fibers more severely compared to minor nerve strains, which may be related to the overexpression of SOCS3 levels in transected injured nerve fibers.^[^ ^40^, ^41^ ^]^ Although no fully regenerative organ exists in the human body, some organs can be restored by dividing and regenerating after injury. Such regenerative capacity is limited, and enhancing the regenerative capacity of tissues can facilitate the development of new therapies and better use of tissue engineering technology. Hence, we have elaborated in the Discussion section that the notable transfection efficiency correlates with the insights presented in Section 2.3: Enhancing the prospects for neural regeneration through genetic engineering emerges as a feasible strategy, necessitating vectors distinguished by their cell‐specific uptake, augmented DNA stability, and adjustable parameters to ensure optimal transfection alongside negligible cytotoxicity.

**Figure 4:**
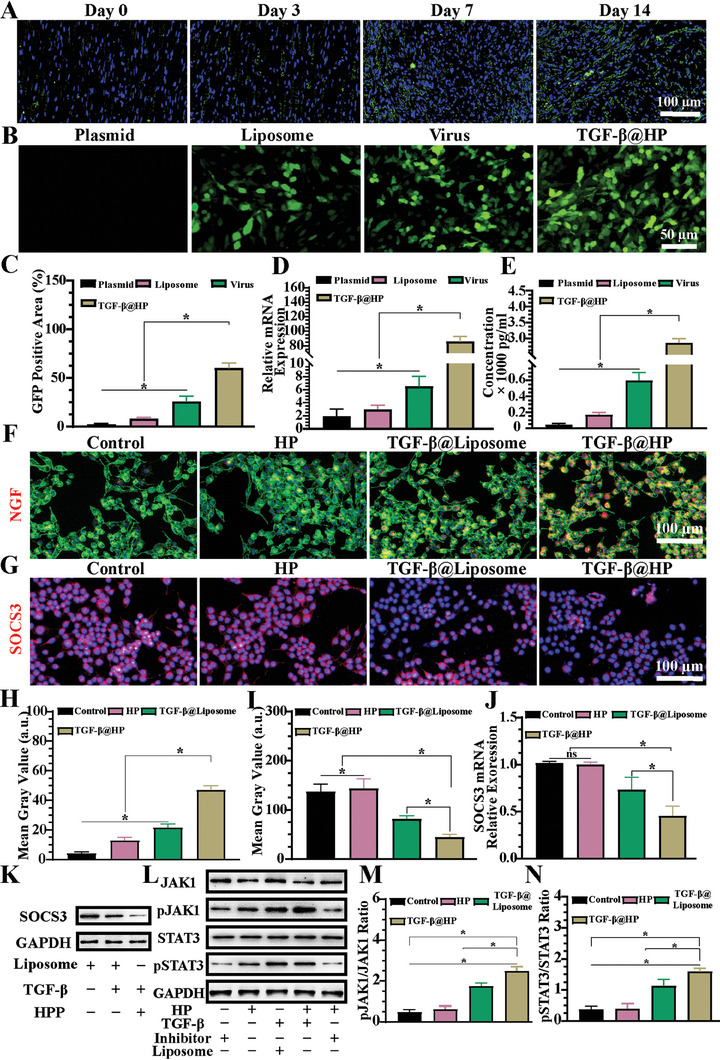
Influence of transforming growth factor‐beta (TGF‐β) on neuronal regeneration in vitro. A) Temporal expression patterns of SOCS3 following neural injury in vivo, as delineated by green fluorescence labeling of SOCS3. B) Introduction of TGF‐β plasmid into cells via liposomes, viruses, and HP hydrogels resulted in the manifestation of green fluorescent protein (GFP), marking the expression of GFP. C) Quantitative analysis of GFP fluorescence intensity was conducted using ImageJ software. D) The alteration in TGF‐β expression post‐transfection was quantified via reverse transcription‐polymerase chain reaction (RT‐PCR). E) The post‐transfection secretion levels of TGF‐β were determined through enzyme‐linked immunosorbent assay (ELISA). F) Changes in nerve growth factor (NGF) expression following TGF‐β transfection, with NGF visualized in red fluorescence, F‐actin in green, and the nucleus in blue. G) The expression levels of SOCS3 were assessed through immunofluorescence, with SOCS3 and the nucleus labeled in red and blue fluorescence, respectively. H) The fluorescence intensity of NGF was statistically analyzed utilizing ImageJ software. I) ImageJ software was also employed to analyze the fluorescence intensity of SOCS3. J) The relative expression of SOCS3 was evaluated via RT‐PCR. K) Protein expression of SOCS3 following TGF‐β transfection. L) Analysis of relative protein expression within the TGF‐β/SOCS3/JAK1/STAT3 signaling pathway. M) Proportion of phosphorylated JAK post TGF‐β transfection was quantified using ImageJ software. N) The extent of phosphorylated STAT3 following TGF‐β transfection was analyzed with ImageJ software. (These observations, derived from independent investigations, are presented as mean ± standard deviation, with *n* = 5; statistical significance is indicated by ★ *p* < 0.05, and nonsignificant outcomes are noted as ns).

The present study further confirms that SOCS3 expression is time‐dependently increased on the peripheral nerve fiber surface following transection injury (**Figure** **4**A). Through the construction of TGF‐β plasmids with GFP green fluorescent protein and transfection of TGF‐β plasmid DNA using Plasmid, Liposome, Virus, and TGF‐β@HP, we found that after 24 h of in vitro incubation, the TGF‐β@HP group showed the most GFP green fluorescence under fluorescence microscopy, which was superior to Liposome and Virus alone transfected. The results were statistically significant (Figure 4B,C). The transfection efficiency was evaluated by examining the expression of TGF‐β after transfection using reverse transcription‐polymerase chain reaction (RT‐PCR) and enzyme‐linked immunosorbent assay (ELISA). We found that the nano hydrogel could be transfected more efficiently than traditional liposome transfection. More levels of TGF‐β were detected within the TGF‐β@HP group, and the results were statistically significant (Figure 4D,E).

Moreover, we evaluated the effect of TGF‐β overexpression in SCs on neural differentiation function. We first assessed the expression of NGF and found that TGF‐β significantly increased the expression level of NGF. The amount of TGF‐β transfection was found to be proportional to the expression level of NGF. This increase in NGF has been widely reported to promote neural differentiation and axonal regeneration (Figure 4F,H). To investigate the potential mechanism of TGF‐β on neural regeneration regulation, we examined the SOCS3 level in cells transfected with TGF‐β and found that TGF‐β could reduce the expression level of SOCS3 in SCs. The expression level was negatively correlated with the concentration of TGF‐β (Figure 4G,I). After transfection with TGF‐β, we examined the expression of related genes (Figure 4J), and its results reveal the same trend. Moreover, the western‐blot (WB) results showed that TGF‐β was negatively correlated with the expression level of SOCS3 (Figure 4K). phospho‐tyrosine‐protein kinase JAK1 (pJAK1) and phospho‐signal transducer and activator of transcription 3 (pSTAT3) were activated after transfection, and the statistical analysis showed that the expression levels of phosphorylated tyrosine‐protein kinase JAK1 (JAK1) and signal transducer and activator of transcription 3 (STAT3) were increased. The JAK1/STAT3 pathway is closely related to neural regeneration and is an essential pathway for the transduction of neural differentiation (Figure 4L–N). It has been widely reported to play an important role in nerve proliferation, differentiation, promotion of axonal growth and guidance, promotion of peripheral nerve regeneration, regulation of neural‐immune response, and providing a favorable physiological environment for nerve regeneration.^[^ ^42^, ^43^ ^]^

### 2.4 Composite Catheters Reduce Reactive Oxygen Species Production and Apoptosis In Vitro

Within 24 h of nerve transection injury, the distal axon ruptures and commences Wallerian degeneration. The axon and its myelin sheath separate, and the remaining nerve fiber axonal structure fractures to create axoplasm.^[^ ^44^, ^45^ ^]^ This substance provides the foundation for nerve regeneration. Axonal degeneration begins in the distal axon and gradually advances to the distally innervated organs and tissues, ultimately resulting in the loss of innervation of the limb's motor and sensory functions.^[^ ^46^ ^]^ Wallerian degeneration is the process of nerve axonal apoptosis. Following degeneration, the fibers forfeit their original autonomous nerve fiber structures, including their anatomical structures and functions, such as the absence of motor and sensory nerve fiber axons.^[^ ^47^ ^]^ The inadequate restoration of function after suturing nerve endings may be attributed to the loss of nerve fiber integrity and independence. While nerve fibers have some regenerative capacity, it is far from sufficient for the process of nerve injury associated with large nerve defects and Wallerian degeneration. Even though Wallerian degeneration provides the material foundation for nerve regenerative processes, it is also an essential obstacle to poor nerve function recovery.^[^ ^48^ ^]^ Therefore, for clinical patients, if nerve fiber postoperative continuity can be reinstated, but their proper motor and sensory innervation cannot be exerted, surgical intervention may prove to be ineffective.

Axonal degeneration occurs primarily due to a local reduction in NAD^+^ levels following nerve axon dissection.^[^ ^49^ ^]^ Inadequate intra‐axonal NAD^+^ within nerve fibers leads to the deprivation of nutritional and metabolic support of the cell body connected to the detached portion of the axon, resulting in a loss of energy supply and protein synthesis capacity at the detached end. This eventually leads to further degeneration of the myelin sheath and axon and the formation of necrotic tissue.^[^ ^50^ ^]^ Some studies have attempted to hinder Wallerian degeneration to restore axonal integrity, such as the local application of NGF to the axon site and the use of non‐steroidal anti‐inflammatory drugs to provide nutritional support, which can, to some extent, delay the onset of Wallerian degeneration.^[^ ^51^, ^52^ ^]^ In our research, we found that the decrease in NAD^+^ and the accumulation of local NMN initiate axonal degeneration. We attempted to increase NAD+ expression at the injury site, alleviate the buildup of local NMN, and rescue axonal degeneration in the hope of sustaining and prolonging the anatomical integrity of the nerve axon.

**Figure 5:**
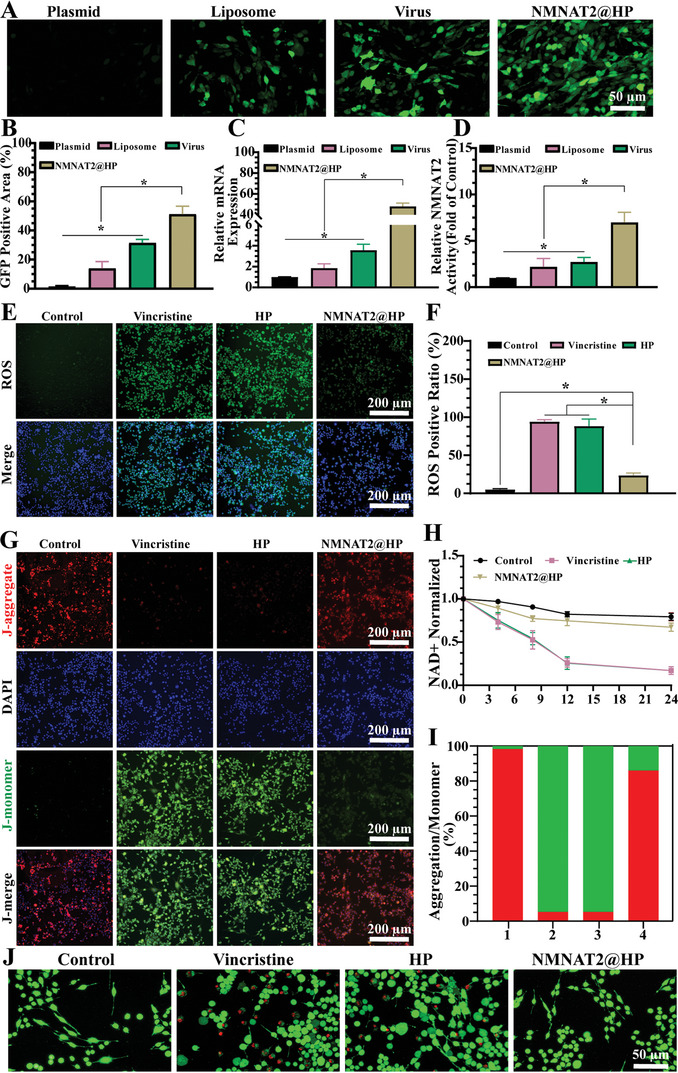
The suppressive influence of NMNAT2 on Wallerian degeneration. A) Introduction of NMNAT2 fused with a GFP tag, evidenced by the green fluorescence indicative of GFP activity. B) Quantitative evaluation of GFP fluorescence was conducted utilizing ImageJ software. C) The presence of NMNAT2 was verified through reverse transcription‐polymerase chain reaction (RT‐PCR). D) Analysis of the enzymatic activity associated with NMNAT2 subsequent to its transfection. E) Assessment of reactive oxygen species (ROS) levels in SCs following NMNAT2 transfection, with ROS denoted by green fluorescence and nuclei by blue fluorescence. F) The intensity of ROS fluorescence was statistically analyzed using ImageJ software. G) Alterations in mitochondrial membrane potential were discerned through JC‐1 staining, with red fluorescence identifying multimers, green fluorescence specifying monomers, and nuclei highlighted in blue. H) The concentration of NAD+ post‐transfection was determined. I) JC‐1 fluorescence, indicative of mitochondrial health, was analyzed statistically (where 1, 2, and 3 represent the control group, the vincristine group, the HP group, and the NMNAT2@HP group, respectively). J) Viability of SCs post‐vincristine treatment was assessed via live‐dead staining, with viable cells marked in green and non‐viable cells in red. (These results, gleaned from independent studies, are presented as mean ± standard deviation, *n* = 5; statistical significance is indicated by ★ *p* < 0.05, with non‐significant findings labeled as ns).

In this investigation, we initially transfected the NMNAT2 enzyme into nerve cells in vitro using nano hydrogels and scaffolds, followed by assessing the transfection efficiency via GFP‐labeled fluorescent and RT‐PCR (**Figure** **5**A–C). The results showed that the scaffold transfection group exhibited greater green fluorescence under the fluorescence microscope. Both statistical and PCR results indicated that more NMNAT2 was expressed in nerve cells, and the enzyme activity of NMNAT2 was detected (Figure 5D). The results demonstrated that the group transfected with HP hydrogel had significantly higher enzyme activity than the other control group, while there was no significant difference in the enzyme activity of the group transfected with TGF‐β@HP scaffold. All the above outcomes were statistically significant.

Vincristine, a widely known drug capable of inducing Wallerian degeneration of nerve cells, was used to intervene with the transfected cells. The results revealed that Vincristine increased reactive oxygen species (ROS) in SCs and produced a large amount of green fluorescence. Nevertheless, increasing the expression level of NMNAT2 could alleviate ROS caused by Vincristine. The statistical results suggested that NMNAT2 delivery by HP hydrogels reduced ROS production, restored normal cell homeostasis, and exhibited no statistical difference compared to the control (Figure 5E,F). Wallerian degeneration is frequently linked to cell apoptosis, and we further evaluated the apoptosis of SC cells. The 5,5′,6,6′‐tetrachloro‐1,1′,3,3′‐tetraethylbenzimi‐ dazolylcarbocyanine iodide (JC‐1) staining results revealed that the alteration of early mitochondrial membrane potential was an indication of early cell apoptosis. After cell injury, the mitochondrial membrane potential decreased, and JC‐1 transformed from red aggregates to green monomers. The staining results displayed that NMNAT2 could mitigate the detrimental effects of vincristine on nerve cells and reduce cell apoptosis. The staining results also showed that the intracellular apoptosis decreased after NMNAT2 transfection, with no statistical difference compared to the blank control group (Figure 5G,I). Additionally, we assessed the level of NAD^+^ in SCs undergoing early apoptosis. In the Wallerian degeneration caused by vincristine, the intracellular NAD^+^ level decreased, but NMNAT2 transfection significantly increased the NAD+ level and maintained intracellular homeostasis (Figure 5H). The outcome of Wallerian degeneration is cell apoptosis, and our live and death staining demonstrated that fewer dead cells were observed after NMNAT2 transfection. These findings indicate that NMNAT2 transfection into cells utilizing gel‐infused scaffolds could reduce cell apoptosis, maintain cell homeostasis, and provide a suitable environment for cell regeneration (Figure 5J and Figure S3, Supporting Information).

### 2.5 Sciatic Nerve Regeneration and Nerve Functional Recovery In Vivo

**Figure 6:**
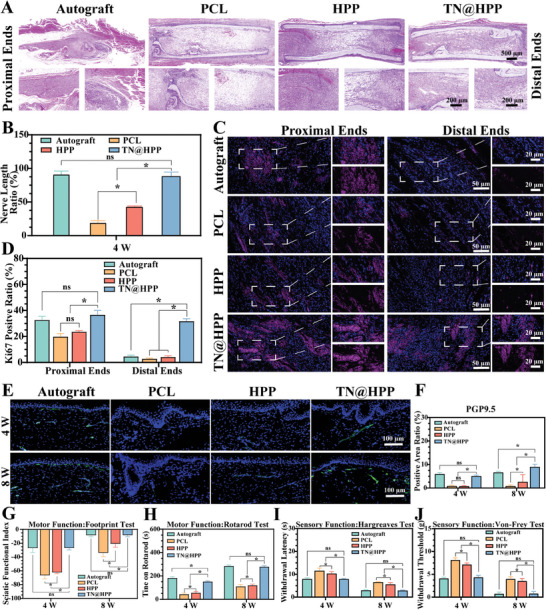
Histological evaluation and functional restoration of the regenerated sciatic nerve. A) Analysis of hematoxylin and eosin (H&E) stained longitudinal sections of regenerated nerves 4 weeks post‐transplantation, including detailed views of both the proximal and distal segments. B) Quantitative assessment of H&E staining was performed using ImageJ software. C) The regenerative potential of proximal and distal nerve fibers was evaluated through Ki67 immunofluorescence staining, highlighting Ki67 in red and nuclei in blue. D) ImageJ software facilitated the statistical analysis of Ki67 staining. E) Investigation of PGP9.5 expression in the plantar skin of the lower extremities, with PGP9.5 protein denoted in green, and quantitative analysis of PGP9.5 immunofluorescence staining conducted using ImageJ software. F) The restoration of motor function in the lower limbs was appraised using the footprint analysis. G) Evaluation of lower limb motor capabilities was further conducted through the rotation test. H) The reinstatement of sensory functions in the skin was determined by the Hargreaves test. I) The recovery of cutaneous sensory abilities was also assessed via the Von Frey test. (These findings, derived from independent studies, are reported as mean ± standard deviation, with *n* = 5; statistical significance is denoted by ★ *p* < 0.05, while non‐significant results are indicated as ns).

Therefore, in our research endeavor, we designed neural conduits through spatially differentiated distribution, which have achieved a state of enhanced nerve regeneration properties at the proximal end while effectively impeding degenerative apoptosis of the nerve structure at the distal end. This transformative approach has effectively resolved the quandary of substantial nerve defects by simplifying it into a readily solvable conundrum of interstitial nerve sutures. By harnessing the potential of spatial structural disparities, we have ensured the existence of a 0.2–0.3 cm interstice, achieving an autonomous selectivity of nerve axon fiber regeneration which aligned with previous clinical studies. This strategy facilitated the harmonious integration of sensory nerve‐sensory nerve and motor nerve‐motor nerve fibers. Anchored upon the aforementioned strategy, our meticulously devised multifunctional gene delivery catheter was surgically implanted into animals afflicted with extensive segmental nerve defects. Histological analysis revealed that implanted NGCs were well biocompatible in rats (Figure S4, Supporting Information). After 4 weeks and 8 weeks, the nerve catheter was carefully retrieved and subjected to tissue sectioning, followed by staining using the H&E method (**Figure** **6**A and Figures S5 and S6, Supporting Information). These staining outcomes allowed for the observation of nerve fiber growth within the nerve catheter, with predominant growth at the proximal end. Notably, even though the dense nerve fibers within the catheter lumen did not achieve the normal structural characteristics of nerve fibers, such satisfactory outcomes were attained within the initial four weeks of nerve repair. Consequently, a statistical analysis was conducted to measure the length of the regenerated nerve fibers within the composite catheter (Figure 6B). The findings revealed that the length of regenerated nerve fibers in the TN@HPP conduit closely corresponded to that observed in the autologous nerve graft group. These results exhibited no significant statistical disparities and proved to be considerably superior to those observed in the PCL‐only and HP groups. Additionally, the regenerative capacity of nerve fibers during the intermediate phase of repair was evaluated. Staining was performed to assess the proliferation capacity of nerve fiber cells at both the proximal and distal ends. The staining outcomes showcased that, at the proximal end, the nerve fibers demonstrated a degree of regenerative capacity, as indicated by substantial positive areas exhibiting Ki67 levels (Figure 6C). The area and fluorescence intensity of these positive areas within the multifunctional nerve conduit group surpassed those observed in the autograft group by a substantial margin. These findings underscore the robust regenerative potential of nerve fibers that had not completed the entire repair process, particularly during the intermediate stage. Moreover, these outcomes surpassed those observed in the PCL and HP groups, with fewer positive areas noted at the distal end and a notable proliferation of cells within the multifunctional nerve conduit group. Statistical analysis of the data yielded significant disparities in the results (Figure 6D).

In the nascent phases of nerve repair, our innovative composite nerve conduit approach has demonstrated auspicious outcomes regarding tissue functionality. What singularly characterizes our composite nerve conduit, distinguishing it markedly from its counterparts, is its dual action: it fosters nerve regeneration at the proximal end while inducing consistent apoptosis at the distal end. Moreover, the unique spatial configuration of our conduit facilitates a “gap suture” mechanism, enabling nerve fibers to self‐align and selectively interconnect, thereby reinstating structural integrity and reestablishing the neural sensory‐motor correspondence. This feature prominently sets our nerve conduit apart in its capacity to singularly enhance nerve regeneration, marking a cornerstone in our research endeavor. Consequently, our investigation extends to evaluating the sensory functions of the dermis in the lower limb, innervated by the sciatic nerve, alongside the motor functions of the muscle tissue, thereby broadening the scope of our inquiry into the reparative potentials of our conduit. Initially, we scrutinized the protein gene product 9.5 (PGP9.5) marker, an indicator of peripheral nerve sensory and motor function, and duly observed significant green fluorescence indicative of PGP9.5 within both the autograft group and the TN@HPP conduit group (Figure 6E). This observation was particularly prominent in the PCL conduit group and the HPP conduit group, resulting in marked discrepancies in their statistical analysis results (Figure 6F). Subsequently, a discrete evaluation of the motor and sensory functions of the lower extremity was undertaken. First, the assessment of motor function was performed through the utilization of the Footprint Test and the Rotarod Test (Figure 6G,H). These tests revealed noticeable progress in motor function restoration during the early four‐axis period and later eight weeks of repair. The recovery of motor function in the lower extremity was strikingly superior to that observed in the standalone PCL control group and the HPP group, with discernible statistical disparities compared to the autograft group. Conversely, the evaluation of nerve sensory function was conducted utilizing the Hargreaves Test and the Von‐Frey Test (Figure 6I,J). The findings demonstrated that the multifunctional composite nerve conduit successfully reinstated the sensory function of the nerve, exhibiting consistent outcomes when compared to the autograft group and significantly surpassing those observed in the PCL group and the HPP group. These results presented notable statistical variances.

### 2.6 Histological Analysis of Regeneration Nerve Fiber

**Figure 7:**
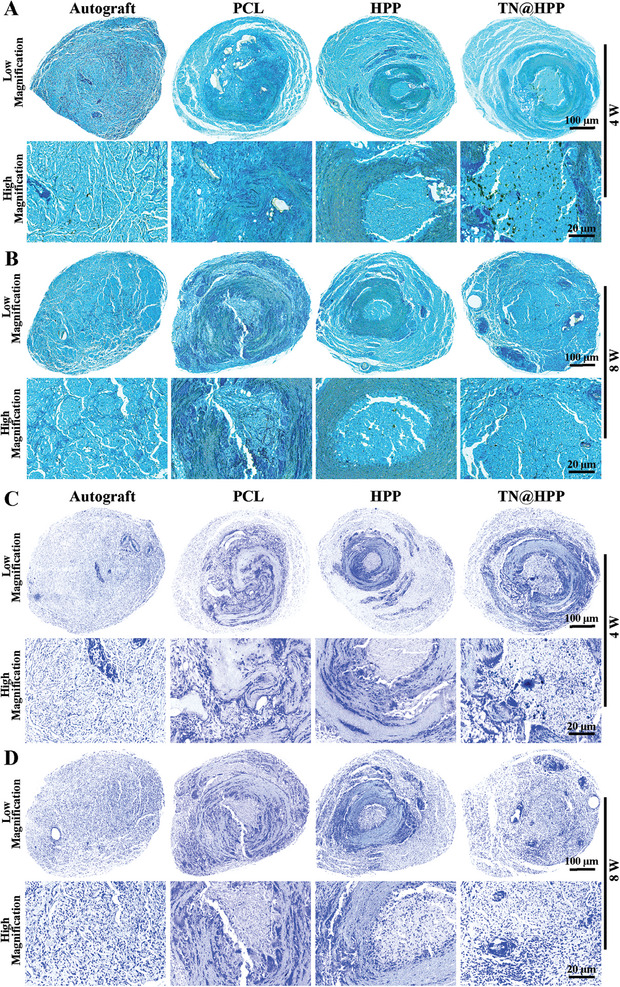
Histological cross‐sectional staining of myelinated nerve fibers. A) Luxol fast blue (LFB) staining depicts the myelinated nerve fibers at 4 and 8 weeks, showcasing both the complete cross‐sectional view and an enlarged detail (B). C) Toluidine Blue (TB) staining illuminates the regenerative journey of nerve fibers at 4 and 8 weeks, presenting a comprehensive cross‐sectional perspective alongside a detailed magnification (D).

Peripheral nerve fibers comprise myelinated and unmyelinated nerve fibers, each fulfilling distinct roles in upholding nerve transmission.^[^ ^53^, ^54^ ^]^ Following nerve injury, demyelination of myelinated nerve fibers occurs, leading to the disruption of their pristine architecture. This process exposes the axons, subsequently subject to degradation by macrophages at the injury site, culminating in the formation of axoplasm.^[^ ^55^ ^]^ This progression, known as Wallerian degeneration, further exacerbates the onset of degeneration. Hence, the evaluation of nerve fiber regeneration necessitates careful consideration of myelin sheath formation and the abundance and ratio of myelinated nerve fibers. To this end, we employed luxury fast blue (LFB) and toluidine blue (TB) staining techniques to visualize the myelin sheaths of the nerves. As illustrated in the figure, at the 4 weeks, discernible lumen‐like structures persisted within the implanted nerves, with blue‐stained myelin sheaths observable within their lumens. In the autograft group, we observed robust nerve fiber formation, characterized by a substantial presence of myelinated nerve fibers (**Figure** **7**A). By week 8, a limited number of residual nerve ducts were visible in both the PCL and HPP groups, with positive myelin formation detected within and around the luminal space. Regarding the TN@HPP conduit group, the majority of the nerve conduit had undergone degradation, while a dense formation of myelin, akin to that seen in autologous nerve grafts, was discernible in the cross‐section of the nerve's middle segment (Figure 7B).

Encouragingly, our TB staining results corroborated these findings. In the autologous nerve graft group, a small quantity of myelinated nerve fiber formation was noted at the 4 weeks (Figure 7C). As repair time progressed, a multitude of densely myelinated nerve fibers became conspicuously apparent after 8 weeks. Conversely, the PLC and HPP groups exhibited a lesser degree of repair during the same timeframe compared to the autologous nerve graft group, implying that the efficacy of simple single‐ or multi‐channel nerve conduits employed in clinical practice still falls short of the gold standard represented by autologous nerve graft repair in animal models. Notably, our TN@HPP conduit group displayed a small number of myelinated nerve fibers at the initial stages of repair (Figure 7D). Consequently, the aforementioned findings highlight the multidimensional nature of our strategy for nerve structure and functional restoration, wherein the formation of myelin and myelinated nerve fibers contribute to functional repair. This multifaceted approach distinguishes our strategy from conventional single‐lumen and multi‐lumen nerve conduits, enabling the attainment of superior repair outcomes.

### 2.7 Compound Nerve Conduits Facilitated the Upregulation of S100B and NF200 in Nerve Fibers

PNI possesses inherent regenerative capacity, although the precise mechanisms and cytokine involvement in this process remain unexplored.^[^ ^56^ ^]^ However, the direct application of neurotrophic factors at the injury site has been demonstrated to enhance nerve fiber regeneration, while the spatiotemporal differential expression of neurocytokines promotes the directed migration and differentiation of nerve fibers. Numerous studies have attempted to incorporate growth factors into NGC to bolster the regenerative capacity at the injury site. While some progress has been made, challenges persist due to the limited availability of neurotrophic factors and the inability to precisely match their concentration and distribution with the spatial variances of nerve dynamic growth. Consequently, certain investigations have employed gene delivery techniques such as Adeno‐associated viruses (AAV) and nanoparticles to transport NGF‐related gene fragments to the injury site, enabling sustained cytokine release over an extended period.^[^ ^57^, ^58^ ^]^ This approach partially alleviates the issue of cytokine deficiency. However, it is accompanied by low transfection efficiency and an inability to generate sufficient concentrations of cytotoxic factors.

**Figure 8:**
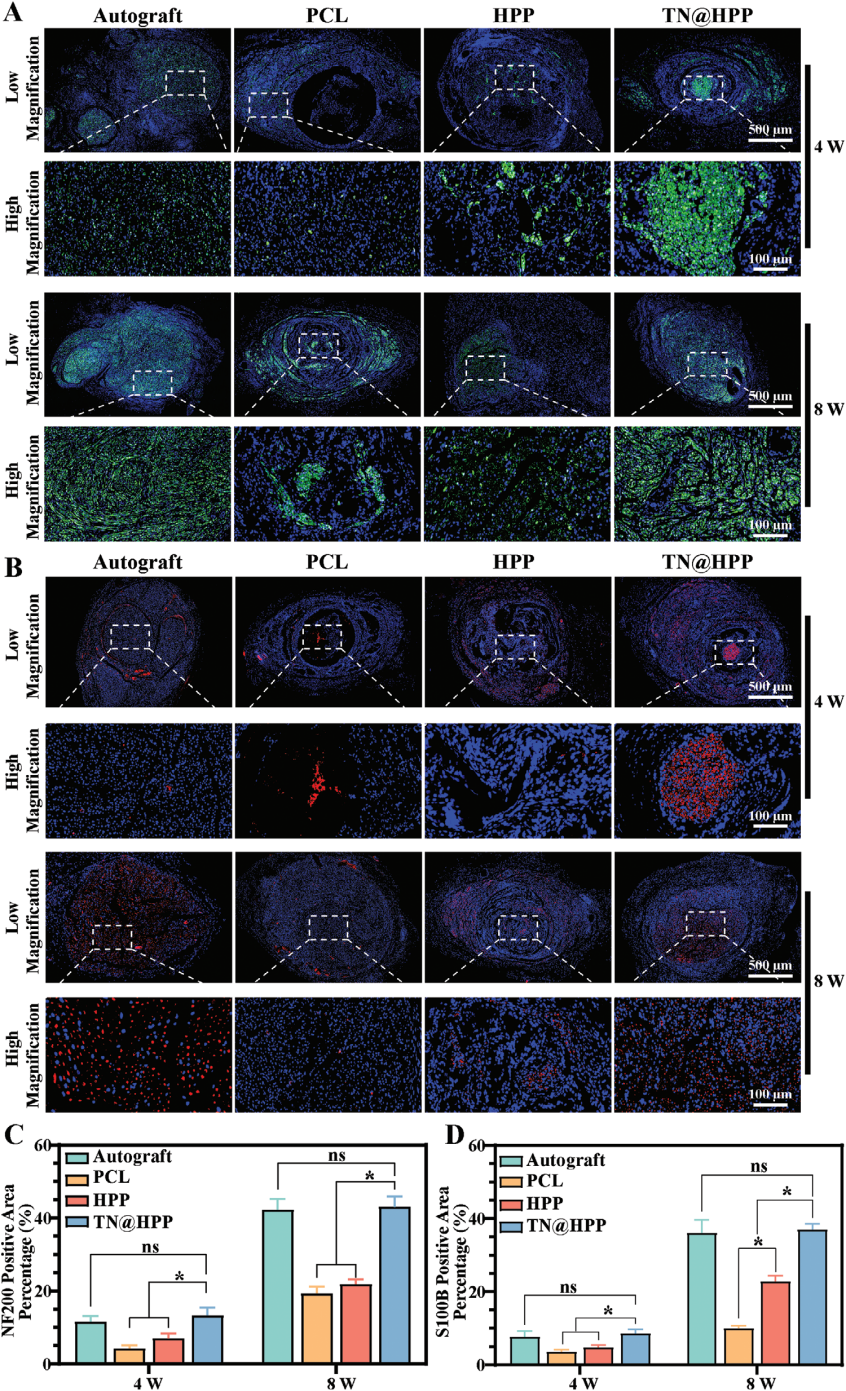
Employment of NF200 and S100B immunofluorescence to evaluate the maturity of regenerating nerve fibers. A) Immunofluorescence staining for NF200 protein in neural tissue, accompanied by images at both low and high magnification, with subsequent quantitative analysis via ImageJ software (C). B) Immunofluorescence delineation of S100B protein within neural tissue, presented through images at varying magnifications and analyzed statistically using ImageJ software D). (These findings, stemming from independent studies, are compiled as mean ± standard deviation, *n* = 5; statistical significance is noted by ★ *p* < 0.05, while findings deemed non‐significant are marked as ns).

To evaluate the efficacy of our composite catheter in nerve repair, we conducted cross‐sectional immunofluorescence staining of regenerating nerve mid‐sections at 4 and 8 weeks. Initially, we assessed the expression levels of NF200, and the observations revealed the presence of green‐labeled nerve fibers within nerve sections (**Figure** **8**A,C). Newborn nerve fibers were also discernible within the catheter, with a progressive increase in nerve axons over time. Furthermore, an expansion in the area of positive green fluorescence was evident in the PCL and HPP nerve catheter groups. However, the degree of nerve regeneration in these groups was comparatively lower than that observed in the TN@HPP conduit group, wherein a substantial NF200 expression was observed within the lumen of the early canal. After 8 weeks of repair, no significant differences in the degree of nerve regeneration were observed between the TN@HPP conduit group and the autologous nerve graft group. Next, we evaluated the expression levels of S100B (Figure 8B,D). Notably, the PCL and HPP groups exhibited expression levels lower than those of the autograft and TN@HPP conduit groups. Moreover, the expression levels at 8 weeks were significantly higher than those at 4 weeks. The reparative potential progressively improved with time.

### 2.8 Functional Recuperation of Innervated Muscles Following Nerve Injury

Nerves primarily execute their functions by transmitting electrical signals from nerve centers to innervate the tissues and organs under their dominion, including muscles, skin, and bone tissues.^[^ ^59^ ^]^ In clinical practice, diverse factors can contribute to suboptimal recovery of nerve function, such as incongruity of severed nerve ends and demyelination changes within the nerves. The assessment of nerve fiber repair and restoration of muscle function can be conducted at the animal level through tissue sectioning and electromyography, respectively. In the clinical realm, nerve conduction is often evaluated by examining the motor and sensory functions of the patient's limbs, occasionally complemented by electromyography.

**Figure 9:**
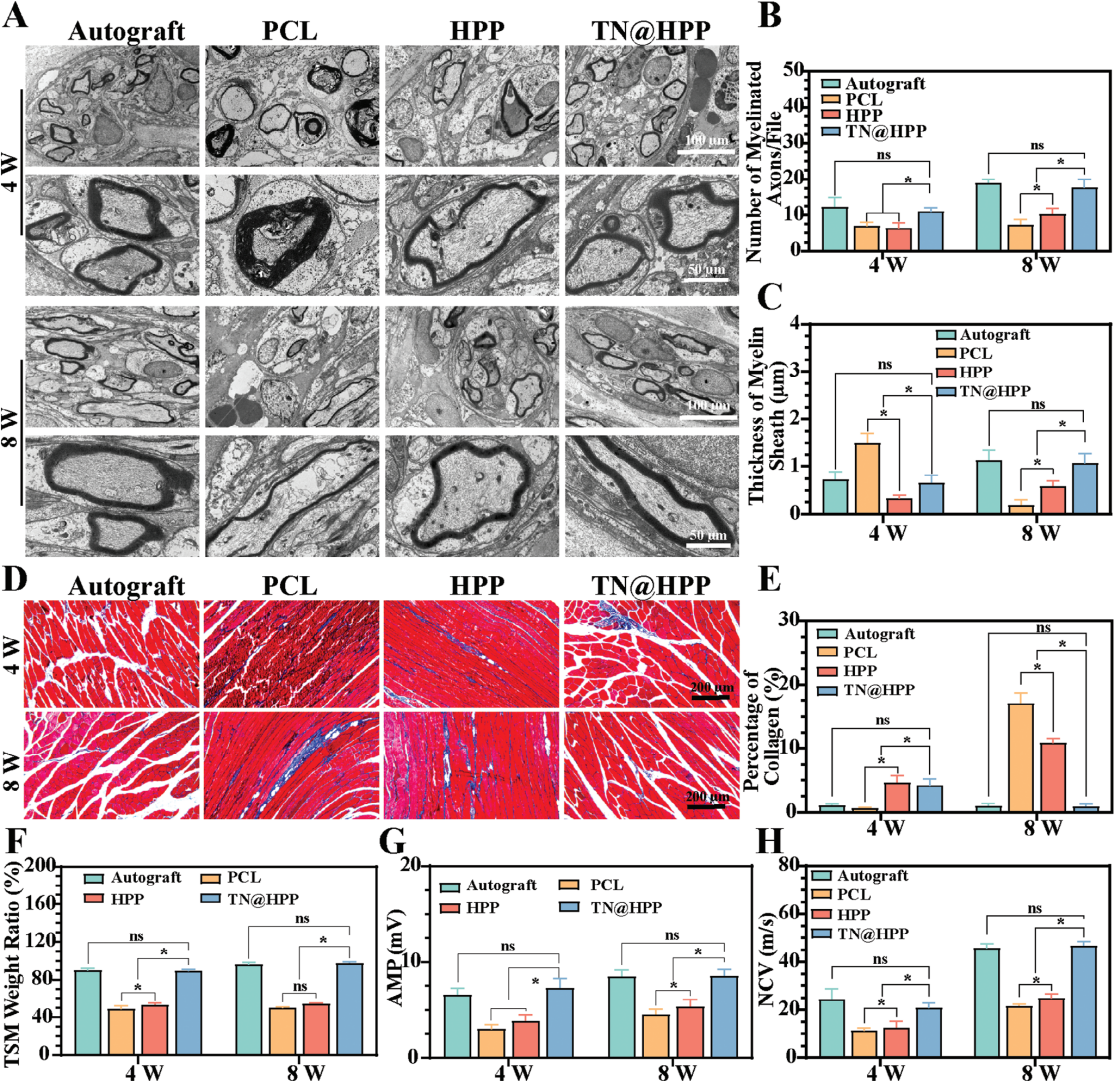
Restoration of functionality in nerve and muscle fibers. A) Transmission electron microscopy (TEM) imagery showcasing cross‐sectional views of nerve fibers. B) Quantification of myelin sheath numbers in regenerated nerves and the assessment of myelin sheath thickness in these nerve fibers C), conducted through statistical analysis with ImageJ software, utilizing TEM images for data acquisition. D) Application of Masson's trichrome staining to the calf triceps muscle (TSM), followed by a quantitative evaluation of its collagen content via ImageJ software E). F) Analysis of the triceps surae muscle's weight as a percentage of total body weight. G) Electrophysiological measurements revealing the amplitude of electrical signal propagation (AMP) and the velocity of nerve conduction (NCV) in nerve fibers, as determined through electromyography at 4 and 8 weeks post‐treatment. (These observations, derived from independent research, are presented as mean ± standard deviation, with *n* = 5; statistical significance is indicated by ★ *p* < 0.05, while non‐significant results are annotated as ns).

In this present study, preliminary findings suggest that animals achieved favorable sensory and motor functional restoration following repair with the composite nerve conduit. Furthermore, the restoration of anatomical integrity within the nerve tissue fibers could be observed through tissue section staining. To align with clinical assessment methods, electromyography, and additional examinations were performed to ascertain the efficacy of nerve repair. Initially, tissue sections from the regenerated nerve tissue's middle segment were stained and subjected to TEM analysis, revealing the presence of black circular myelin tissue (**Figure** **9**A). Disruption of the myelin sheath structures on the PCL and HPP indicated inadequate nerve repair. Statistical analysis results demonstrated significant variations in the thickness and quantity of nerve myelin sheaths across different nerve conduit groups after nerve injury. The autologous nerve graft group and the TN@HPP conduit exhibited greater structure and quantity of myelin sheaths in the regenerated nerves compared to the other two control groups (Figure 9B,C). Subsequently, the triceps calf muscle innervated by the sciatic nerve was assessed through sectioning and weighing (Figure 9D). Tissue section staining indicated damage to the muscle fibers in the PCL and HPP groups, characterized by collagen formation and the loss of their dense fibrous structure. Statistical analysis revealed comparable muscle recovery between the autologous nerve graft group and the TN@HPP group, both of which significantly outperformed the other two control groups (Figure 9E). Furthermore, weighing the triceps calf muscle confirmed muscle atrophy and weight loss within the PCL and HPP groups, whereas no significant difference was observed between the autograft and TN@HPP conduit groups, indicating a parallel recovery in muscle function (Figure 9F). Finally, nerve conduction function was evaluated following nerve catheter implantation using electromyography testing. Both AMP and NCV results demonstrated that the PCL catheter and HPP catheter implantation failed to fully reinstate nerve conduction function, while the TN@HPP conduit achieved nerve conduction function comparable to the standard of autologous nerve grafts, yielding statistically distinct outcomes (Figure 9G,H).

## 3 Experimental Section

### 3.1 Materials

The fabrication of the composite conduits is demonstrated in the schematic (Figure 1). Both poly (ε‐caprolactone) (PCL) and polyethyleneimine (PEI, branched 600, purity ≥ 99%) were purchased from Sigma‐Aldrich Trading Co., Ltd. (Shanghai, China). Glycidyl methacrylate was grafted to HA to acylate the HA (HA‐GMA) as our research previously.^[^ ^60^ ^]^ TGF‐β and NMNAT2 plasmids were designed and manufactured by Sangon (Sangon Biotech, Shanghai, China). SCs for experiments in vitro were obtained from the cell bank of the Institute of Biochemistry and Cell Biology (Shanghai, China). A cell counting kit (CCK‐8) was obtained from Sigma‐Aldrich Trading Co., Ltd. (Shanghai, China). Hexafluoroisopropanol (HFIP, purity ≥ 99.56%) was obtained from Shanghai Darui Fine Chemicals Co., Ltd. (Shanghai, China). Unless otherwise specified, all the above reagents were used directly. TGFβ‐IN‐2 (Compound 9d) were purchased from the MedchemExpress (USA). All the materials were used as received, except those mentioned otherwise.

### 3.2 Preparation of Nerve Conduits

The TN@HPP composite nerve conduit was synthesized in three sequential steps. First, 0.8 g of PCL was dissolved in 10 mL HFIP forming an 8% (w/v) clarified and homogeneous electrospinning pre‐solution, respectively. The 10 mL of the above prepared PCL/HFIP solution were electrospun to generate nanofibers with a constant speed of 1.5 mL h^−1^ and a voltage of 8 kV. The as‐electrospun fibers were collected onto a stainless steel bar (2.0 mm diameter, 100.0 mm length, rotated at 200 rpm) located 14 cm from the capillary to form fiber tubes, named PCL. Then fiber tubes were vacuumed in a desiccator for 48 h to remove residual HFIP.

Subsequently, for the preparation of TGF‐β pDNA or NMNAT2 pDNA loaded hybrid HA‐GMA/PEI hydrogel (HP hydrogel): Both synthetic and freeze‐dried HA‐GMA matrix and PEI were blended with a relative mass fraction *W*_PEI_/*W*_HA‐GMA_ of 4% and then dissolved in 10 mL double distilled water formed a mixture with 3% concentration. After stirring until clarified, quantitative TGF‐β pDNA and NMNAT2 pDNA were added to the above‐clarified mixture forming a hybrid solution with a relative mass fraction (*W*_pDNA_/*W*_HA‐GMA/PEI_) of 0.5%, respectively.

For the preparation of TGF‐β pDNA or NMNAT2 pDNA loaded hybrid nerve conduits: HP hydrogel combined with the TGF‐β plasmid was injected into the proximal 8 mm cavity along with the distal 2 mm cavity containing the NMNAT2 plasmid. The proximal 8 mm cavity was filled with HP hydrogel containing the TGF‐β plasmid, while the distal 2 mm cavity was filled with the NMNAT2 plasmid. Last, the hybrid nerve conduits were subjected to freeze‐drying to create multifunctional composite nerve conduits, named TN@HPP. To better evaluate the advancement of TN@HPP, HPP was also prepared. The above‐prepared nanofibers tubes or hydrogel were hybridized to generate nerve conduits according to the parameters in **Table** **1**, respectively.

**Table 1:** Materials usage of PCL fibers tube, HP hydrogel, TGF‐β pDNA, and NMNAT2 pDNA in control and experimental groups, respectively. (+ represent the raw materials for preparing conduits; – represent the unused raw materials for preparing conduits).

Samples	PCL fibers tube	HP hydrogel	TGF‐β pDNA	NMNAT2 pDNA
PCL	+	−	−	−
HPP	+	+	−	−
TN@HPP	+	+	+	+

### 3.3 Characterization of the Conduits

Observation of the visage and morphology of the hollow electrospinning nerve conduit: A camera was employed to document the gelation of diverse constituent gels based on HA‐GMA under the illumination of UV rays, while meticulously recording the time of gel formation. A rheometer was utilized to discern the rheological properties of the hydrogels. The malleability and injectability of the gels were appraised through visual depictions of the gelation process. A universal testing machine was employed to ascertain the force released overtime during the compression cycle of the hydrogel. The morphology of the desiccated hydrogels was scrutinized using a scanning electron microscope, with the pore size and porosity being calculated from the intricate morphology.

Preparation of hydrogel samples: A specific mass of desiccated hydrogel sample was meticulously prepared and its mass (referred to as m0) was meticulously recorded. The mass of the sample was weighed immediately after achieving sufficient water absorption (referred to as m1). Calculate the rate of water absorption and swelling: Employ the following formula to calculate the rate of water absorption and swelling: Water absorption and swelling rate (%) = [(*m*1 – *m*0) / *m*0] × 100. Moreover, in the same way, the change in dissolution diameter was calculated

Detection of plasmid release using UV spectrophotometry (Thermo Fisher Scientific, USA): In vitro, degradation was determined by first meticulously weighing the lyophilized hydrogel (M0) and then placing it in DTT and PBS (Gibco, USA) at 37 °C respectively. The hydrogel was subsequently lyophilized and weighed at a specific time point (*M*_t_). Degradation rate (%) = (*M*_0_ – *M*_t_) / *M*_0_ × 100%.

The different constituents of hydrogels were individually perfused into PCL catheters, and the cross‐sectional morphology of the composite catheters was scrutinized using scanning electron microscopy. Subsequently, the mechanical properties of the axial drawing of the infused hydrogel conduits were meticulously tested using a universal testing machine.

### 3.4 Cell Culture

SCs were obtained from the cell bank of the Institute of Biochemistry and Cell Biology (Shanghai, China) to evaluate the impact of composite conduits on nerve regeneration. The SCs were cultivated on the nanofibers using high‐glucose DMEM (Gibco, USA) complete medium, supplemented with 10% fetal bovine serum (Gibco, USA) and 1% penicillin‐streptomycin (Servicebio, Wuhan, China). The SCs were cultured at a temperature of 37 °C in a CO_2_ culture incubator.

### 3.5 The Biocompatibility of the Composite Conduit

The meticulously prepared PCL electrospun film sheets were initially immersed into diverse constituents of hydrogels to craft composite film sheets (PCL‐Mats: PCL electrospun film sheets in their singular state; HP‐Mats: film sheets soaked in the HA‐GMA/PEI gel; TN@HPP‐Mats: film sheets immersed in the HP gel infused with TGF‐β and NMNAT2 plasmids), thereafter, these distinct film sheets were situated within the culture plate, and the cells were nurtured upon the membrane sheets.

#### 3.5.1 CCK‐8

The morphology of SCs on the nanofibers was observed via SEM (PCL‐Mats, HP‐Mats, TN@HPP‐Mats). A quantity of 1.0 × 10^5^ cells was cultured on the electrospun fibers for 48 h (PCL‐Mats, HP‐Mats, TN@HPP‐Mats), fixed with 2% glutaraldehyde, and subsequently observed by TEM after undergoing dehydration. The proliferation of cells on different fibers was examined using the cell count kit‐8 (CCK‐8) (Beyotime Biotechnology, China). Initially, 2.0 × 10^3^ cells were seeded on nanofiber films, placed in 96‐well plates, and incubated in an incubator for 1, 3, and 5 d. The CCK‐8 kit was then added and incubated for 30 min, followed by the measurement of absorbance changes using a multifunctional enzyme marker.

#### 3.5.2 Living/Dead Test

The viability of cells on the electrospun fiber films was assessed using living and dead cell staining (Beyotime Biotechnology, China) (PCL‐Mats, HP‐Mats, TN@HPP‐Mats). A total of 5.0 × 10^4^ SCs were initially seeded on electrospun fiber films in 24‐well plates and incubated for 48 h. Subsequently, calcium and PI were added, incubated for 15 min in the incubator, and observed and scanned under a confocal microscope. The surviving live cells were represented by the color green, while the deceased cells were represented by the color red.

### 3.6 The Migration of SCs on Nanofibers

To evaluate the migration of SCs, 5.0 × 10^4^ cells were seeded in the upper chambers and cocultured with various nanofibers in the lower chamber (PCL‐Mats, HP‐Mats, TN@HPP‐Mats). After 48 h of coculturing, the migrated cells were stained with crystalline violet, and images were captured using an inverted microscope (Leica, Germany). The results were analyzed using Image J software3.6 for the immunofluorescence staining of the SCs. The Transwell is purchased from Corning Company (USA).

### 3.7 Immunofluorescence Staining

A population of 5.0 × 10^4^ cells was seeded onto the electrospun fibers and cultured in the incubator for 24 h. The cells were then subjected to three washes with PBS and subsequently fixed in a 4% paraformaldehyde solution for 30 min. After another round of PBS washing, the cells were incubated in an immunostaining blocking solution (Beyotime Biotechnology, China) for 1 h to facilitate closure. Next, rabbit‐derived NGF monoclonal primary antibody (Abcam, Cambridge, UK) and rabbit‐derived SOCS3 monoclonal primary antibody (Abcam, Cambridge, UK) were added and allowed to incubate overnight at 4 °C in a refrigerator. Following a thorough wash, RBITC and CY3‐labeled secondary antibodies (Beyotime Biotechnology, China) were separately introduced. After another round of washing, FITC‐labeled peptide (Beyotime Biotechnology, China) was added and allowed to incubate for 40 min to label the cell membrane. Finally, DAPI (Beyotime Biotechnology, China) was added and incubated for 5 min to enable observation of protein expression under a fluorescence microscope (Leica, Germany). Images were captured for subsequent calculation of protein expression levels.

### 3.8 Transfection of TGF‐β and NMNAT2

The transfection efficiency of plasmid gene fragments was evaluated through the expression of GFP fluorescence using a hydrogel‐infused catheter. Initially, cells were seeded at a density of 2.0 × 10^4^ per well in 24‐well plates and allowed to incubate for 24 h. Once the cells reached 70%–80% confluence, both sides were washed with opti‐MEM serum‐free medium (Thermo Fisher, USA), after which the cells were individually transfected according to the steps for liposome transfection of cells. In the case of liposome transfection, 2.5 µg of pDNA and 5 µL of liposomes (Thermo Fisher Scientific, USA) were combined and added to the cells for a duration of 12 h. Subsequently, the cells were washed with PBS and provided with a fresh complete medium. For viral transfection, the adeno‐associated virus (Sangon Biotech, Shanghai, China) was chosen as the vector, and the transfection plasmid was inserted into the viral vector to construct the transfection plasmid. This plasmid was then amplified and purified for transfection, with an equal amount of viral transfection vector directly added to the cells for culture. Regarding hydrogel transfection of pDNA, cells were cultured as described earlier, washed with opti‐MEM medium, and exposed to 100 µg per mL of hydrogel (containing 2.5 µg of pDNA). After 12 h of transfection, all cells were provided with a fresh complete medium, and the transfected cells were employed for subsequent experiments. The transfection efficiency was evaluated based on the intensity of GFP fluorescence and RT‐PCR (EZBioscience, China).

### 3.9 RT‐PCR and WB

RT‐PCR: The cells were isolated through grinding in a lysis reagent, and subsequently, 1 µg of total RNA was reverse transcribed using the Revert First Strand cDNA Synthesis Kit (EZBioscience, US). A reaction volume system was established, consisting of 20 µL of SYBR Green Master Mix, 1.6 µL of primers, 4 µL of cDNAs, and 14.4 µL of H_2_O, all by the manufacturer's instructions of the SYBR Green Master Mix (EZBioscience, USA). The mRNA internal reference utilized was 3‐phosphoglycerate dehydrogenase (GAPDH). The 2^−ΔΔCT^ method was employed to determine relative gene expression. The results were obtained from three replicates. The primer was purchased from the Servicebio company (China), the primer sequence were obtained from Table S1 (Supporting Information).

WB: Western blotting procedures were carried out as described in a previous study. Following the intervention, the cells were harvested and lysed in RIPA buffer (Sigma, Chian). Total protein was extracted and its concentration was measured using the BCA Protein Assay Kit (Beyotime Biotechnology, China). The proteins were separated by electrophoresis on a 10% SDS‐PAGE gel at 110 V for 1 h and then transferred to 5% fat‐free milk for blocking. Subsequently, the samples were sequentially incubated with primary antibodies and secondary antibodies. Enhanced chemiluminescence reagents (Thermo Fisher Scientific) were utilized for imaging. Primary antibodies against SOCS3, JAK1, pJAK1, STAT3, and pSTAT3 were obtained from Abcam (Cambridge, UK).

### 3.10 ELISA

TGF‐β concentrations were determined using the R&D ELISA kit, specifically the TGF‐β Quantikine HS ELISA Kit (from R&D Systems, Minneapolis, MN), following the manufacturer's instructions. SCs (2.8 × 10^4^ cells cm^−2^) were seeded on plates and incubated for a designated period. After treatment with 200 µL (100 µg mL^−1^) of nanospheres, the plates were further incubated for 3 d. The supernatant was collected and subjected to ELISAs as instructed. The absorbance was measured using a multi‐detection microplate reader (BioTek, USA) and quantified accordingly.

### 3.11 Mitochondrial Membrane Potential Assessment through JC‐1 Staining

The cells were cultured in 24‐well plates with 2.5 × 10^4^ cells per well and transfected according to the specified groups and transfection procedures. Subsequently, the changes in mitochondrial membrane potential were examined by introducing vincristine intervention for 24 h. Initially, the JC‐1 staining working solution (Beyotime Biotechnology, China) was prepared following the instructions. After adding 500 µL of the staining solution and thorough mixing, the cells were incubated in the incubator for 20 min. Following the incubation, the cells were washed with JC‐1 washing solution, and the mitochondrial membrane potential was observed under a fluorescence microscope after replacing the medium with a fresh complete medium.

### 3.12 ROS Detection

The cells were grown in 24‐well plates with 2.5 × 10^4^ cells per well and transfected according to the designated groups and transfection steps. Subsequently, the levels of ROS were examined by introducing vincristine intervention for 24 h (Beyotime Biotechnology, China). The DCFH‐DA staining solution was diluted with serum‐free culture medium at a ratio of 1:1000 to achieve a final concentration of 10 µmol L^−1^. The cell culture medium was removed, and 500 µL of DCFH‐DA staining solution was added. The cells were incubated at 37 °C in a cell incubator for 20 min. Afterward, the cells were washed three times with serum‐free cell culture medium to remove any unabsorbed DCFH‐DA, and the level of ROS was observed under fluorescence microscopy (Leica, Germany).

### 3.13 NAD^+^ Measurement

The cells were transfected in 24‐well plates with 2.5 × 10^4^ cells per well, following the grouping and transfection steps described earlier. Subsequently, NAD+ levels were assessed by introducing permethrin intervention for 24 h. The axons and cell bodies were lysed with 5 m perchloric acid. The resulting extracts were centrifuged, and the supernatant was collected. It was then neutralized with 3 m K_2_CO_3_ and diluted in potassium phosphate buffer. NAD+ was determined using high‐performance liquid chromatography (HPLC) on an LC‐18T HPLC column (Supelco) at a flow rate of 1 mL min^−1^. The elution peaks were matched to the NAD+ standard (Beyotime Biotechnology, China).

### 3.14 Animal Implantation In Situ

All the procedures were duly approved by the Animal Care and Experimental Committee of West China Hospital, Sichuan University (ethical approval number: 20230111003). Male Sprague‐Dawley (SD) rats weighing between 150 and 200 g were acquired to establish a large segmental defect model (1 cm) in the right sciatic nerve, and the repair of nerve regeneration was conducted using electrospun fiber catheters. A total of 72 SD rats were randomly assigned to four groups, each comprising 18 animals: i) autografts, ii) PCL, iii) HA‐GMA/PEI/PCL (HPP), and iv) TN@HPP. Following the methodology described in previous research, the rats were anesthetized to expose the right sciatic nerve after a 10 mm resection. Subsequently, a catheter of equal length but different composition was implanted. In the autograft group, the resected nerve was inverted and sutured between the severed ends of the nerve at 4 and 8 weeks after the surgery, respectively. Pentobarbital was administered for pain relief, and the sciatic nerve tissue at the surgical site was stained for subsequent histological analysis.

### 3.15 Histological and Morphological Evaluation of Regenerated Nerve

#### 3.15.1 Histological Evaluation

Sections of the regenerated sciatic nerve were stained and analyzed at 4 and 8 weeks postsurgery, focusing on the longitudinal and mid‐segment regions. Initially, the nerve tissue was collected for fixation, subjected to a gradient dehydration process, and then embedded in paraffin. Subsequently, staining was performed using hematoxylin and eosin (H&E), toluidine blue (TB), and luxury fast blue (LFB) solutions (Servicebio, China). Finally, the sections were observed under a microscope (Leica, Germany), and images were captured.

#### 3.15.2 Immunofluorescence Staining

Paraffin sections, prepared as described above, were deparaffinized and incubated with anti‐NF200 (1:100, Abcam, UK) antibody and anti‐S100B (1:100, Abcam, UK) antibody, respectively. The sections were incubated overnight, and rinsed, and cell nuclei were labeled using DAPI (Servicebio, Wuhan, China). Observation and image acquisition of the sections were conducted using a Leica fluorescence microscope.

### 3.16 Transmission Electron Microscopy

Freshly obtained nerve tissues were fixed with 2.5% glutaraldehyde (Servicebio, China) and stored overnight in a refrigerator at 4 °C to preserve the nerve's microstructure. Nerve axons were then prepared into sections and incubated with lead citrate and uranyl acetate. Finally, images were captured using a JEM‐2200FS instrument (JEOL, Japan) for in‐depth neuromorphological analysis.

### 3.17 Motor and Sensory Recovery

#### 3.17.1 Skin Innervation

Immunofluorescence sections were obtained from the right plantar skin of postoperative rats at 4 and 8 weeks. Paraffin sections were prepared as previously described, followed by overnight incubation with PGP9.5 primary antibody (1:100, Abcam, UK). Subsequently, the sections were labeled with DAPI for nuclei (Servicebio, Wuhan, China), and nuclear data were collected using Leica's fluorescence microscope for observation.

#### 3.17.2 Footprint Test

Functional recovery was assessed using the aforementioned reference. The rats were allowed to immerse the soles of their feet evenly in ink paint or dye and then freely walk. After the animal had covered a certain distance, it was removed, and its footprints or footprint patterns were observed and recorded. The gait and locomotor ability of the animal were evaluated by measuring characteristics such as stride length, footprint area and spacing, experimental toe spread (ETS), normal toe spread (NTS), experimental toe height (ELS), and normal toe height (NLS). The sciatic Functional Index (SFI) was calculated using the following formula: SFI = [(ETS‐NTS)/NTS]×100‐[(ELS‐NLS)/NLS]×100.

#### 3.17.3 Rotarod Test

The rats were placed on a platform attached to a rotating pole, and the speed of the rotating pole was gradually increased until the animal was unable to maintain balance and fell. The time at which the animal fell was recorded, and its balance and coordination were assessed based on this time.

#### 3.17.4 Hargreaves Test

The rat was placed on a clear glass plate, and an infrared lamp was focused on the hind paws. A timer was used to record the time it took for the animal to respond to the heat source, thereby assessing its nociceptive sensitivity.

#### 3.17.5 Von Frey

Von Frey fibers of various thicknesses were prepared and sequentially applied to the hind limbs of the rats. Starting with low stimulation force, the force was gradually increased until the mice exhibited a pain response. The thickness and weight of the Von Frey fibers at which the mice displayed a pain response were recorded.

### 3.18 Histological Evaluation of the Triceps Surae Muscle and Electromyography Testing

Masson staining of the triceps surae muscle (TSM): Sections of the TSM were stained with Masson staining and analyzed at 4 and 8 weeks postoperatively. The TSM tissue was harvested and fixed, followed by staining with Masson solutions (Servicebio, Wuhan, China). The collagen ratio was calculated using ImageJ software.

#### 3.18.1 TSM Weight

The TSM was collected at different time points, and the weight of both the implanted and normal limbs of each rat was measured. The TSM weight ratio was calculated as follows: TSM weight ratio (%) = TSM (weight of implanted leg)/TSM (weight of normal leg).

#### 3.18.2 EMG

The functional recovery of the sciatic nerve was assessed using minimally invasive electromyography to examine nerve conduction properties. After anesthesia, the sciatic nerve was dissected and exposed under the EMG probes. The amplitude and nerve conduction velocity were recorded to evaluate the recovery of the nerve fibers.

### 3.19 Statistical Analysis

For statistical analysis, each group contained five parallel samples. Statistical analyses were performed using GraphPad Prism 8, with one‐way and two‐way ANOVA and two‐way ANOVA for statistical analysis. The resulting *P* values (*P < 0.05, ns, *P* > 0.05) were considered statistically significant.

## 4 Conclusions

In this study, we introduce a novel and efficacious platform for the delivery of genes aimed at the restoration of sensory and motor functionalities following injuries to the peripheral nerves, leveraging spatial variances. Through the application of electrostatic spinning technology, we crafted PCL nerve conduits featuring a vacuous architecture. Into these conduits, we selectively infused advanced NMNAT2 and TGF‐β gene delivery hydrogels, namely HP hydrogels, creating TN@HPP composite hydrogel conduits. This design emulates the intricate structural foundation and the nuanced secretion of cytokines inherent in the regeneration and repair processes of peripheral nerves. We strategically manipulated axonal growth both in vivo and in vitro—encouraging axonal regeneration proximally, while concurrently averting axonal Wallerian degeneration distally. Structurally, our efforts culminated in the development of a model for a substantial segmental defect in the rat sciatic nerve, bridged with a 1‐mm gap suture to optimally harness the intrinsic selectivity of axonal regeneration. We have meticulously delineated the pivotal role of these composite conduits in nerve repair and regeneration across multiple dimensions, including gene delivery, cytology, histology, and behavioral analysis. Consequently, this investigation posits the expansion of nerve regeneration‐centric gene delivery methodologies into the realm of tissue engineering as a markedly superior approach for the amelioration of peripheral nerve injuries.

## Conflict of Interest

The authors declare no conflict of interest.

## Supporting information

Supporting Information

## Data Availability

The data that support the findings of this study are available from the corresponding author upon reasonable request.
