# Supplementary material for: Highly Bioadaptable Hybrid Conduits with Spatially Bidirectional Structure for Precision Nerve Fiber Regeneration via Gene Therapy
Source: Adv Sci (Weinh). 2024 Mar 14;11(19):2309306. doi: 10.1002/advs.202309306 (PMC11109652; doi:10.1002/advs.202309306)
Supplement: Supplementary file 1 — Supporting Information [file ADVS-11-2309306-s001.pdf]

ADVANCED SCIENCE
Open Access

Supporting Information

for *Adv. Sci.*, DOI 10.1002/advs.202309306

Highly Bioadaptable Hybrid Conduits with Spatially Bidirectional Structure for Precision Nerve Fiber Regeneration via Gene Therapy

Renliang Zhao, Xiangtian Deng, Jizhao Dong, Chen Liang, Xiaozhong Yang, Yunfeng Tang, Juan Du, Zilu Ge, Dong Wang, Yifan Shen, Lianghua Jiang, Wei Lin, Tonghe Zhu* and Guanglin Wang**

Highly Bioadaptable Hybrid Conduits with Spatially Bidirectional Structure for Precision Nerve Fiber Regeneration via Gene Therapy

*Renliang Zhao [a, g 1], Xiangtian Deng [a, g 1], Jizhao Dong [b], Chen Liang [b], Xiaozhong Yang [a, g], Yungfeng Tang [c], Juan Du [b], Zilu Ge [a, g], Dong Wang [a, g], Yifan Shen [e], Lianghua Jiang [f], Wei Lin [d, *], Tonghe Zhu [b, *], Guanglin Wang [a, g *]*

[a] Orthopedics Research Institute, Department of Orthopedics, West China Hospital, Sichuan University, Chengdu 610041, P.R. China

[b] Multidisciplinary Centre for Advanced Materials, Institute for Frontier Medical Technology, School of Chemistry and Chemical Engineering, Shanghai University of Engineering Science, 333 Longteng Rd., Shanghai 201620, P.R. China

[c] Head & Neck Oncology Ward, Cancer Center, West China Hospital, Cancer Center, Sichuan University, Chengdu 610041, P.R. China

[d] Department of Gynecology, West China Second Hospital, Sichuan University, Chengdu 610041, P.R. China

[e] Spine lab, Department of Orthopedic Surgery, The First Affiliated Hospital, Zhejiang University School of Medicine, Hangzhou 310003, China

[f] Department of Orthopedic Trauma, The First People's Hospital of Kunshan affiliated with Jiangsu University, Suzhou, Jiangsu, 215300, PR China

[g] Trauma medical center, Department of Orthopedics surgery, West China Hospital, Sichuan University, Chengdu 610041, China.

corresponding author: E-mail addresses: linwei@scu.edu.cn (W. Lin), zhutonghe89@163.com (T. Zhu), wglfrank@163.com (G. Wang).

[1] These authors contributed equally to this work

Results

1. the structure of the plasmid DNA

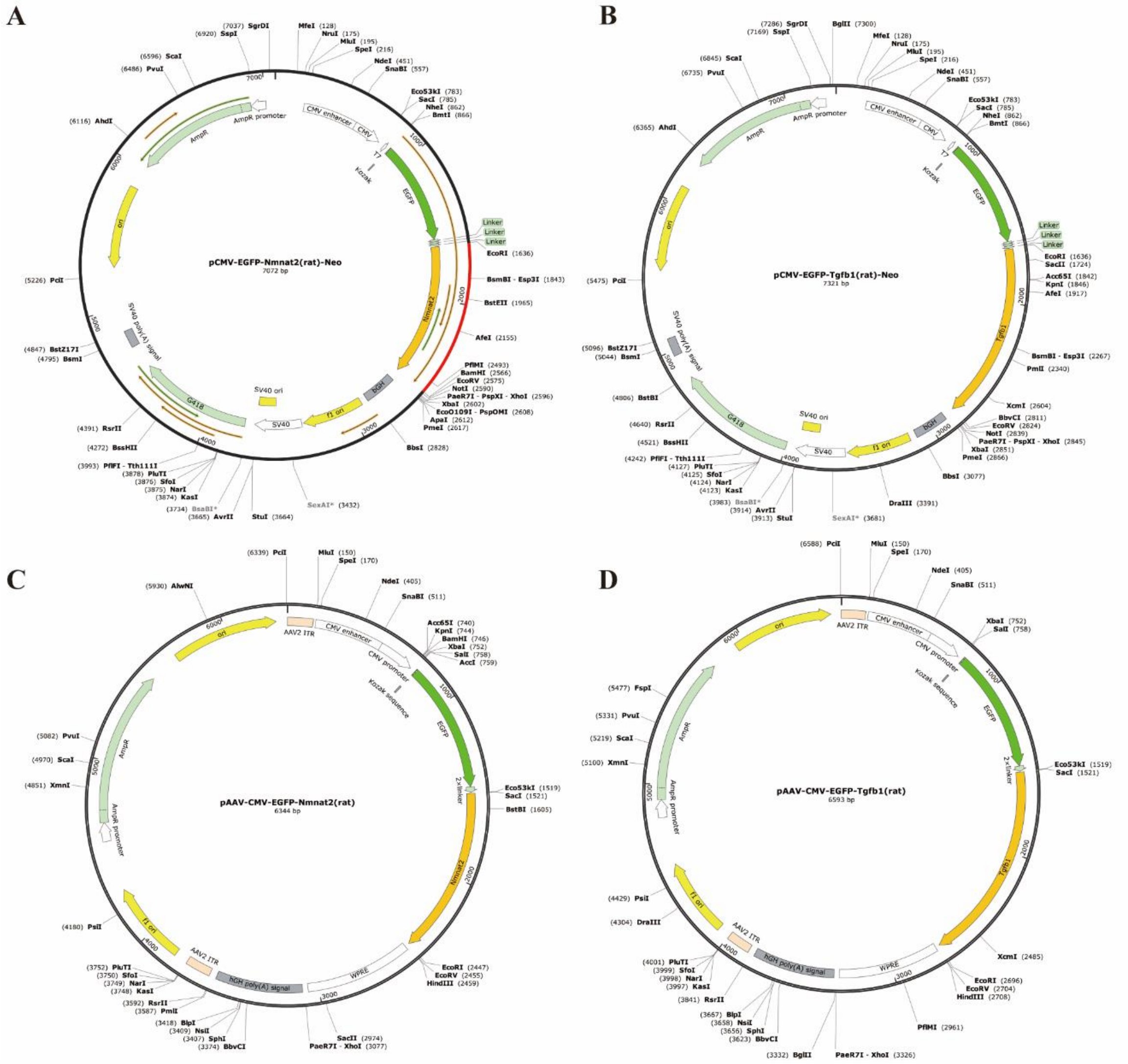

Figure S1. (A) The structure of pCMV-EGFP-Nmnat2. (B) the structure of pCMV-EGFP-Tgfb1. (C) the structure of pAAV-CMV-EGFP-Nmnat2. (D) the structure of pAAV-CMV-EGFP-Nmnat2.

2. the stastatic analysis of NGF fluorescence

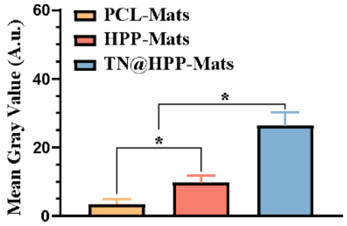

Figure S2. The stastatic analysis of immunofluorescence staining of the NGF in Figure 3G.

3. the stastatic analysis of Live/Dead staining

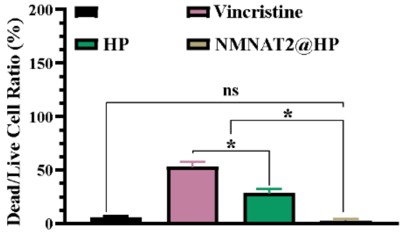

Figure S3. The stastatic analysis of Live/Dead staining in Figure 5J.

4. The biocompatible of the NGC in vivo

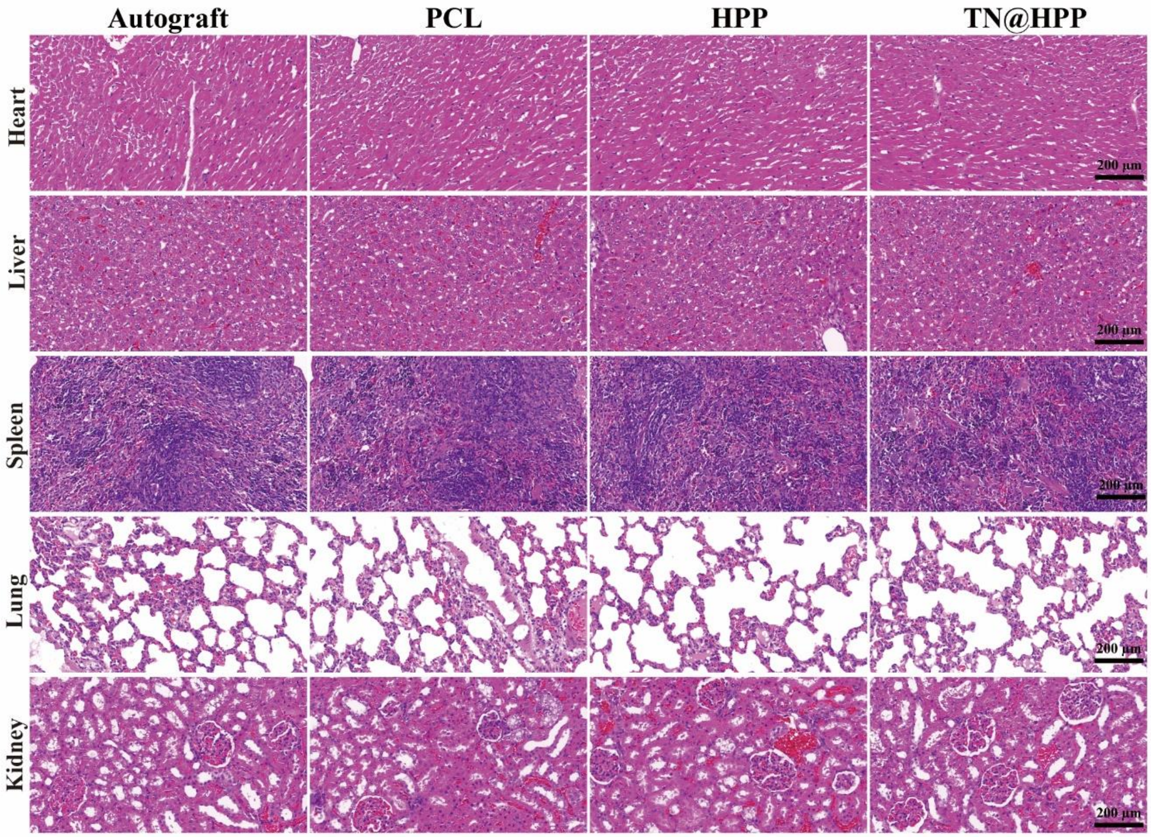

Figure S4. H&E staining of tissue sections of heart, liver, spleen, lungs, and kidneys

5. H&E staining of the sciatic nerve at 8 weeks and its statistical analysis

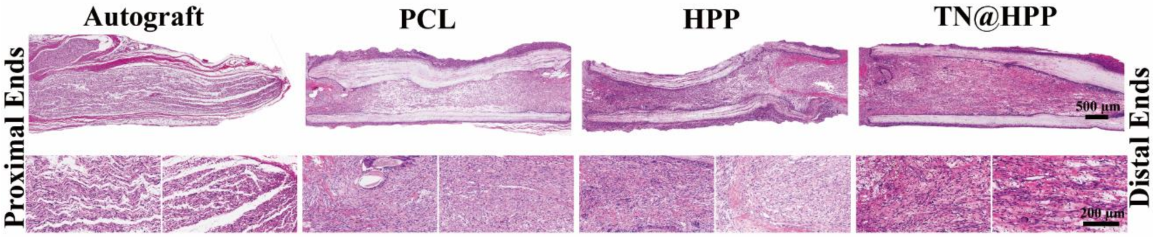

Figure S5. H&E staining of the sciatic nerve at 8 weeks

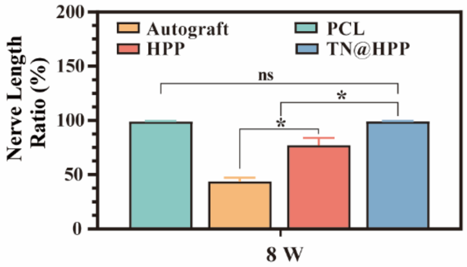

Figure S6. The statistical analysis of H&E staining of the sciatic nerve at 8 weeks

6. the primer of the related genes

Gene	Forward primer	Reverse primer
TGF-β	GCTGAACCAAGGAGACGGAATA	GCAGGTGTTGAGCCCTTTCC
SOCS3	TCACCCACAGCAAGTTTCCC	GCACTCCAGTAGAATCCGCTC
NMNAT2	AATCACTCCTCAATACTCCGCAA	CTGGGACAGGTAATCCACAACG

Table S1. the primer of the related genes
